# Alternative splicing: the pledge, the turn, and the prestige: The key role of alternative splicing in human biological systems

**DOI:** 10.1007/s00439-017-1790-y

**Published:** 2017-04-03

**Authors:** L. M. Gallego-Paez, M. C. Bordone, A. C. Leote, N. Saraiva-Agostinho, M. Ascensão-Ferreira, N. L. Barbosa-Morais

**Affiliations:** Instituto de Medicina Molecular, Faculdade de Medicina, Universidade de Lisboa, Lisboa, Portugal

## Abstract

Alternative pre-mRNA splicing is a tightly controlled process conducted by the spliceosome, with the assistance of several regulators, resulting in the expression of different transcript isoforms from the same gene and increasing both transcriptome and proteome complexity. The differences between alternative isoforms may be subtle but enough to change the function or localization of the translated proteins. A fine control of the isoform balance is, therefore, needed throughout developmental stages and adult tissues or physiological conditions and it does not come as a surprise that several diseases are caused by its deregulation. In this review, we aim to bring the splicing machinery on stage and raise the curtain on its mechanisms and regulation throughout several systems and tissues of the human body, from neurodevelopment to the interactions with the human microbiome. We discuss, on one hand, the essential role of alternative splicing in assuring tissue function, diversity, and swiftness of response in these systems or tissues, and on the other hand, what goes wrong when its regulatory mechanisms fail. We also focus on the possibilities that splicing modulation therapies open for the future of personalized medicine, along with the leading techniques in this field. The final act of the spliceosome, however, is yet to be fully revealed, as more knowledge is needed regarding the complex regulatory network that coordinates alternative splicing and how its dysfunction leads to disease.

## Introduction

Consider a magic trick, one that spans millions and millions of years, performed by a world-class magician known as the spliceosome. From a single gene, multiple RNA products emerge. The results are intriguing: some of these transcripts are almost identical, and others are so unique as to exert antagonising functions. However, the trick is straightforward: it is a simple unit rearrangement of the gene sequence. However, how is such a simple trick performed? Let us unravel the magic of alternative splicing.

Alternative splicing (AS) was first reported in 1977 by the laboratories of Richard Roberts and Philip Sharp, who observed that mammalian cells infected with adenovirus 2 in lytic stage produce mRNA sequences complementary to non-contiguous DNA segments, as confirmed by electron microscopic visualisation of these alternative transcripts hybridised with single-stranded fragments of the viral genome (Berget et al. 1977; Chow et al. 1977). In the following year, Walter Gilbert suggested naming the segments included in and excluded from the mature mRNAs as “exons” and “introns”, respectively (Gilbert 1978).

Splicing in endogenous genes was revealed in the beginning of the 1980s with the findings of calcitonin and immunoglobulin alternative transcripts in mammals (Liu et al. 1980; Tucker et al. 1980; Early et al. 1980a; Rosenfeld et al. 1981, 1982). The contrasting levels of calcitonin expression in rat medullary thyroid carcinoma lines were discovered to be related with alternative transcripts later observed to originate from the same gene and to encode different proteins (Rosenfeld et al. 1981, 1982).

**Fig. 1:**
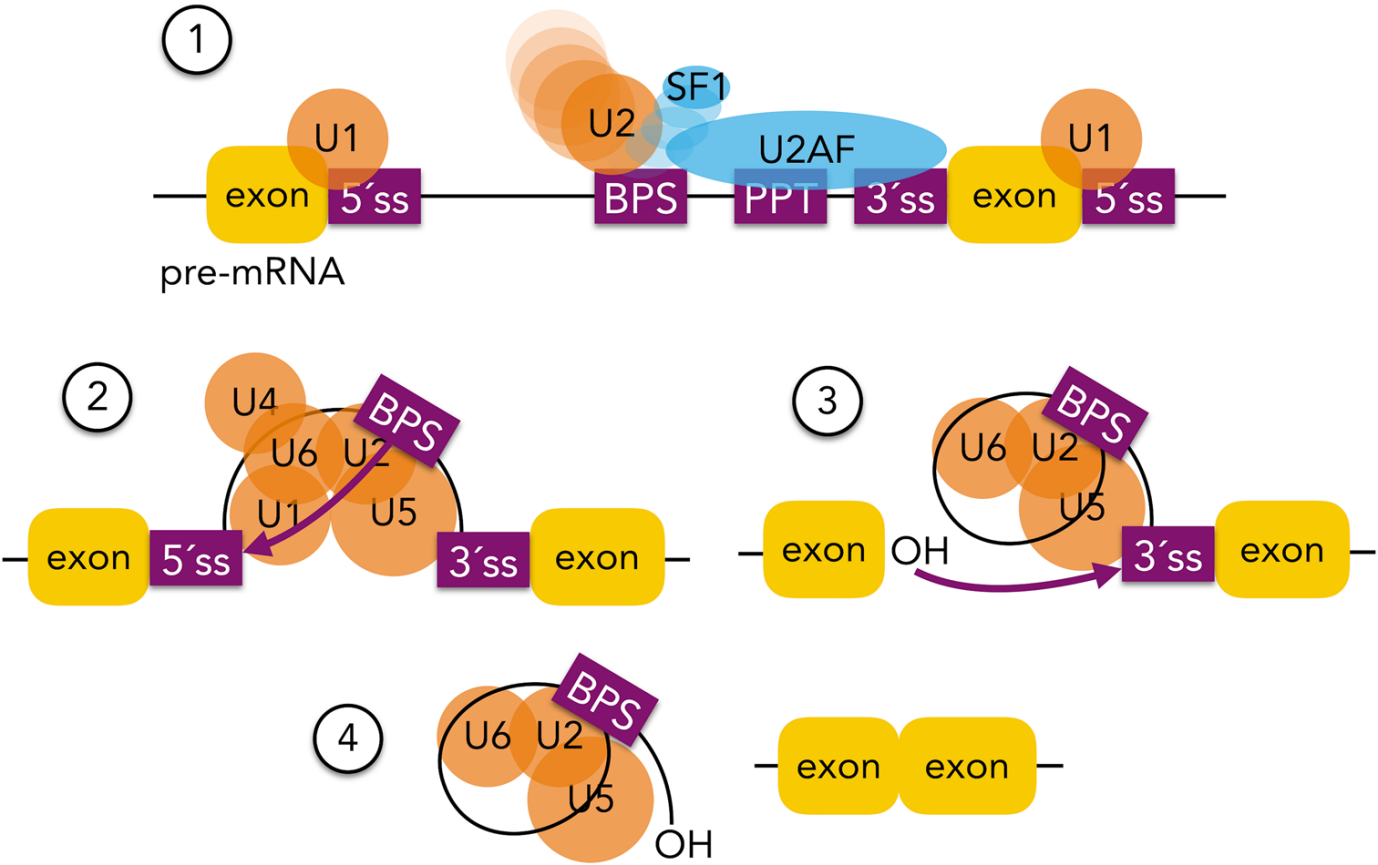
Spliceosome assembly and splicing reactions. (*1*) U1 snRNP binds to the 5′ splice site (5′ss), whereas the splicing factor 1 (SF1) and U2AF proteins bind to the branch point site (BPS), the polypyrimidine tract (PPT), and 3′ splice site (3′ss). The interaction between U1 and U2 snRNPs results in the formation of the pre-spliceosome. (*2*) The first splicing reaction is performed after the recruitment of the U4/5/6 snRNPs through a nucleophilic attack from the adenosine in the BPS to the 5′ss of the upstream exon. (*3*) The intron lariat is then formed. The free 3′ hydroxyl group performs a nucleophilic attack to the phosphate of the 3′ splice site of the downstream exon. (*4*) Finally, the intron lariat is released and both exons are ligated

In addition, in the early 1980 s, the interplay between pre-mRNAs and the U1, U2, U4, U5, and U6 small nuclear ribonucleoproteins (snRNPs) started to be discussed (Lerner et al. 1980; Ohshima et al. 1981; Krainer and Maniatis 1985). These snRNPs are core components of a large ribonucleoprotein complex required for pre-mRNA splicing, known as the spliceosome (Brody and Abelson 1985; Butcher and Brow 2005), that recognises introns through *cis* elements present at exon–intron boundaries (5′ and 3′ splice sites) and within introns (branch point sequence and polypyrimidine tract) (Reed and Maniatis 1985; Chiou and Lynch 2014; Wongpalee and Sharma 2014). As first detailed in 1984, pre-mRNA splicing starts with the spliceosome-catalysed cleavage of the phosphodiester bond at the 5′ exon–intron junction (5′ splice site) performed by a branch point adenosine. This reaction forms an intermediary lariat structure that is subsequently liberated by the cleavage of the phosphodiester bond at the 3′ exon–intron junction (3′ splice site) performed by the free hydroxyl group of the 5′ exon, resulting in the joining of the two exons (Ruskin et al. 1984; Padgett et al. 1984; Domdey et al. 1984; Wongpalee and Sharma 2014) (see Fig. 1).

**Fig. 2:**
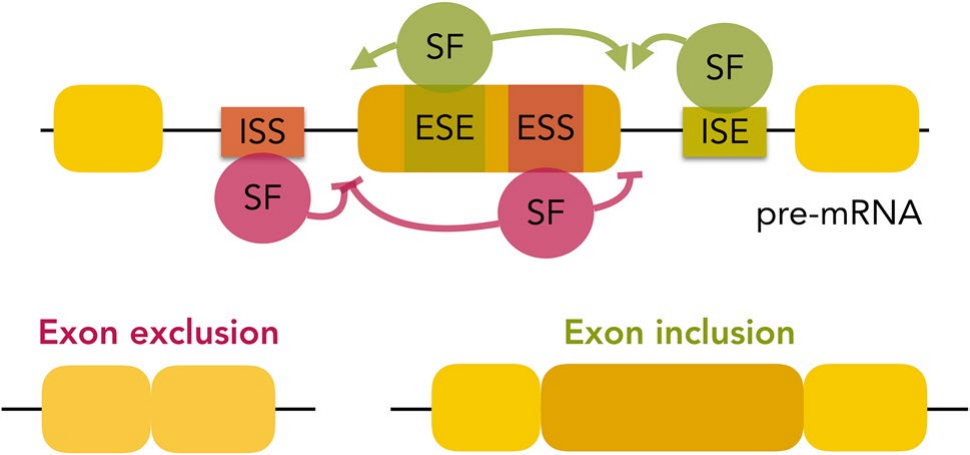
AS regulation by RNA-binding splicing factors. Binding of specific splicing factors (SF) to intronic or exonic splicing enhancers (ISE and ESE, respectively) promotes the inclusion of the alternative exon, whereas binding of given splicing factors to intronic or exonic splicing silencers (ISS and ESS, respectively) inhibits the splicing of the alternative exon

AS is regulated through exonic and intronic *cis*-*acting* regions called exonic/intronic splicing enhancers or silencers that are targeted by RNA-binding proteins (RBPs) (Coelho and Smith 2014). These have been described as *trans*-acting splicing regulators and they have been divided in heterogeneous nuclear ribonucleoprotein particles (hnRNPs, Gallinaro et al. 1981), serine-arginine-rich proteins (SR proteins)—for instance, SRSF1 (Krainer and Maniatis 1985; Krämer and Keller 1985; Krainer et al. 1990), and auxiliary proteins—such as SF1 (Krainer and Maniatis 1985) and U2AF (Ruskin et al. 1988). Those RBPs may promote or inhibit AS or even present opposing regulatory activity depending on their binding sites’ location, as illustrated in Fig. 2 (Goren et al. 2006; Coelho and Smith 2014). Other factors that are relevant for alternative transcripts to be produced include the relative location of *cis* elements, the secondary structure of the pre-mRNAs, sequence modifications (such as those resulting from RNA editing), and epigenetic changes (DNA and RNA methylation, chromatin structure and histone modifications, RNA interference, etc) (Coelho and Smith 2014). The molecular mechanisms of spliceosomal assembly and pre-mRNA splicing are further detailed in the following reviews: (De Conti et al. 2013; Chiou and Lynch 2014; Coelho and Smith 2014; Matera et al. 2014; Sperling 2016).

AS may occur in different manners: exon skipping, intron retention, mutually exclusive exons, alternative first and last exons, alternative 5′ and 3′ splice sites, and alternative “tandem” 5′ and 3′ untranslated regions (UTRs) (Wang et al. 2008; Wagner and Berglund 2014). However, this strict categorisation of AS events may not allow to capture the landscape of more complex AS events (Sammeth et al. 2008).

More recently, the ever-improving development and economical feasibility of genome and transcriptome sequencing have facilitated experiments providing novel insights into the physiological relevance of AS across species and tissues. Such experiments have revealed a higher number of alternatively spliced genes and AS events per gene in birds and mammals when compared to taxonomic groups with fewer cell types, suggesting a link between AS and complexity (Chen et al. 2014). Vertebrates indeed display a tissue-dependent regulation of AS splicing (Blencowe 2006; Barbosa-Morais et al. 2012; Merkin et al. 2012) and 86–88% of human protein-coding genes are reported to undergo AS (Wang et al. 2008; Chen et al. 2014).

AS promotes transcriptome diversity and is reported to be responsible for autophagy and apoptosis regulation and changes in transcription factors, protein localisation signals, protein domains (for instance, binding domain changes that alter protein interactions) and enzymatic properties (such as inactivation or activity modulation of the enzymatic core), among others (Kelemen et al. 2013; Paronetto et al. 2016). In the same line of evidence, several functions appear to be compromised upon dysregulation of AS in multiple human diseases (Tollervey et al. 2011; Oltean and Bates 2014; Paronetto et al. 2016), as a possible result of changes in *cis* (for instance, through mutations or single nucleotide variants—SNVs) or *trans*-acting regulatory elements (through alterations in their expression or protein structure, also potentially caused by SNVs) (Cartegni et al. 2002).

Aside from AS, there are other transcriptional and post-transcriptional mechanisms that regulate gene expression, such as RNA editing and RNA interference. Particularly in primates, a common element between some of these regulation mechanisms is Alu elements, the most abundant transposable sequences in humans (Häsler and Strub 2006; Jeck et al. 2013). Alu elements contain cryptic splice sites that promote exonisation and are reported to more commonly become flanking alternative exons than constitutive exons (Lev-Maor et al. 2008; Jeck et al. 2013). Moreover, intronic Alu elements may regulate AS by shifting splicing patterns through secondary structure changes to pre-mRNAs. These Alu sequences are also usual targets for RNA editing, reported to modify the conserved splice sites required for intron recognition in pre-mRNAs (Rueter et al. 1999). Interestingly, the knockdown of the RNA-editing ADAR1 enzyme in human cells leads to a significant upregulation of circular RNA expression (Ivanov et al. 2015) that compete with AS for the spliceosome recruitment, as this is required for circular RNA formation (Ashwal-Fluss et al. 2014).

The identification of genome-wide RNA–protein interactions, along with RNAi screens (Moore et al. 2010), have allowed to study splicing-regulatory networks associated with specific RBPs through high-throughput sequencing of RNA isolated by crosslinking immunoprecipitation (HITS-CLIP, also known as CLIP-Seq) (Licatalosi et al. 2008) or higher resolution, single-nucleotide CLIP-based techniques followed by high-throughput sequencing, such as iCLIP-Seq (Rossbach et al. 2014) and PAR-CliP (Hafner et al. 2010). These technologies allow to sequence RNAs targeted by an RBP of interest and have already been used to map, in mouse brains, the RNA–protein-binding sites of key splicing regulators such as Nova (Licatalosi et al. 2008), Rbfox (Weyn-Vanhentenryck et al. 2014), and Ptbp2 (Licatalosi et al. 2012). For instance, Ptbp2 has been shown to inhibit multiple adult-specific alternative exons in murine brains (Licatalosi et al. 2012), and more recently, it has been reported that the exclusion of exon 9 in human *PTBP2*-paralog *PTBP1* alters the regulatory activity of approximately 1500 AS events (Gueroussov et al. 2015).

These and other mechanisms of regulation of AS have been extensively studied in different physiological and disease contexts, revealing an additional level of tissue-specific post-transcriptional control characterised by tight spatio-temporal modulation of gene expression. This review will elaborate on the critical contribution of the AS program to the high levels of transcriptomic complexity and functional specificity in human development and physiology. Moreover, it approaches AS changes in the context of host–pathogen interactions, neurodegeneration, cancer, and other pathological conditions. Finally, it concludes with a discussion on therapeutic approaches targeting AS.

## Alternative splicing in the nervous system

The human brain contains over a trillion neurons that are connected to each other through highly specific and convoluted patterns of synaptic connections (Zaghlool et al. 2014). Due to its intrinsic complexity, the mammalian nervous system has evolved to generate a vast protein diversity by the extensive use of AS (Ule et al. 2006). In fact, AS is more abundant in the brain, comparing with other organs (Barbosa-Morais et al. 2012). Moreover, the nervous system has a specific way of regulating its AS programme based on the observations that some RBPs are uniquely expressed in neuronal populations, suggesting that they may control cell type and synapse-specific functions (Traunmüller et al. 2016). Therefore, AS in the brain must be tightly regulated, since the slightest change in splicing outputs can have profound effects in several important neuronal aspects such as neurogenesis and synaptic function (Lipscombe and Diane 2005).

**Fig. 3:**
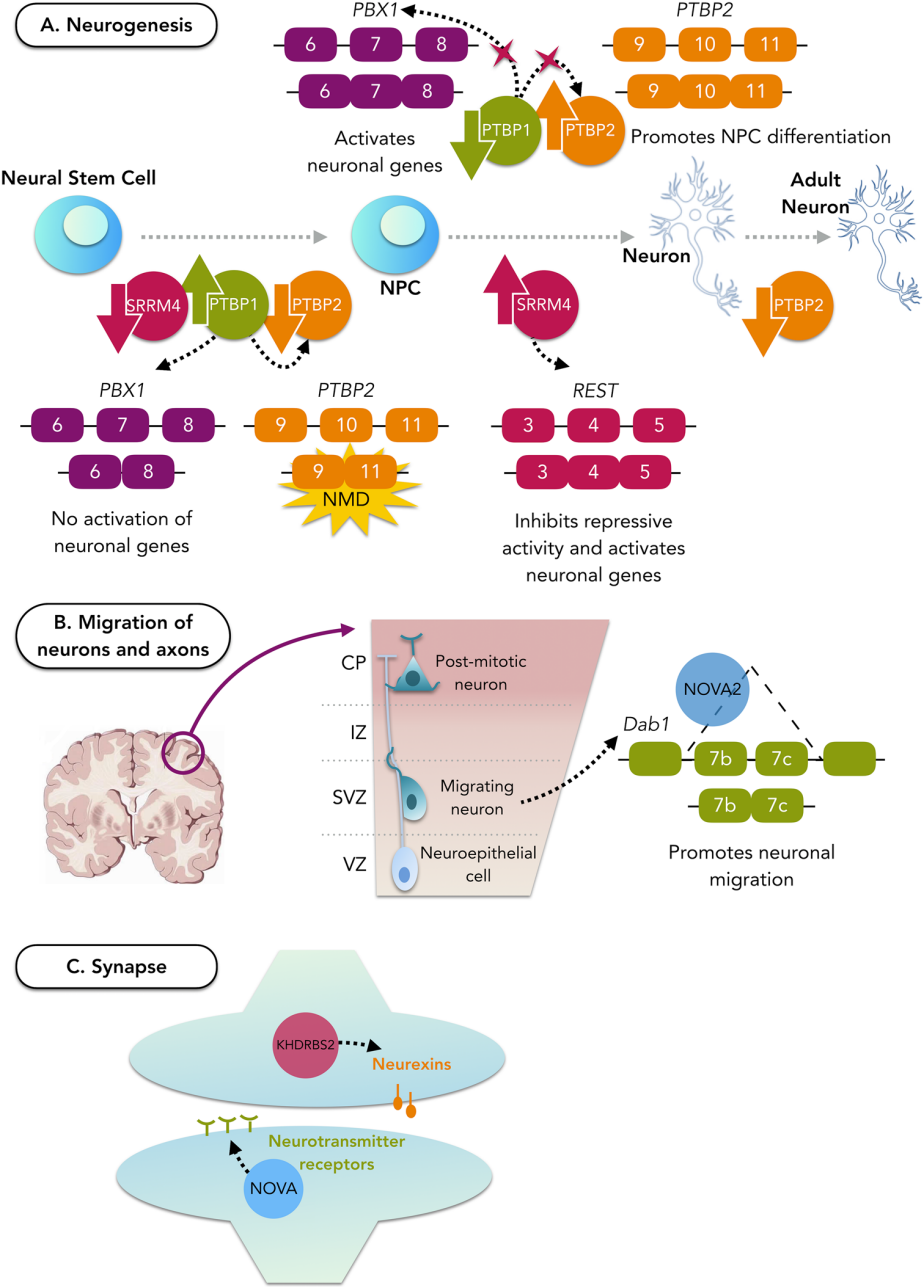
Role of splicing factors during neurogenesis and neuron maturation. *A* PTBP1 is responsible for repressing the activation of neuronal genes and is highly expressed in neuronal stem cells and neuronal progenitor cells. Upon differentiation, PTBP1 becomes downregulated, allowing the induction of PTBP2 and PBX1 that will activate neuronal genes. SRRM4 also becomes expressed during neuronal differentiation and contributes to it by inactivating REST, a repressor of activation of neuronal genes. After the neurons become mature, the levels of PTBP2 decrease, giving rise to an adult neuronal splicing programme. *NMD* nonsense-mediated decay, *NPC* neural progenitor cell. *B* Once the neuronal cell fate commitment is achieved, neurons can migrate to generate the laminar structure of the brain. NOVA2 is a splicing factor particularly important for the cortical lamination since it regulates AS of Dab1 to promote neuronal migration. *VZ* ventricular zone, *SVZ* subventricular zone, *IZ* intermediate zone, *CP* cortical plate. *C* For the maturation process, neurons form synapses. This process is equally controlled by splicing factors, namely, KHDRBS2 that regulates neurexins (presynaptic cell-adhesion proteins), which are essential for synapse formation and transmission, and the NOVA family that regulates AS of neurotransmitter receptors

During neurogenesis, AS patterns vary considerably. In the onset of neuronal differentiation, these switches of patterns are mainly regulated by changes in the expression of PTB (polypyrimidine tract binding) proteins, namely, PTBP1 and PTBP2, and SRRM4 (serine/arginine repetitive matrix protein 4) (Raj et al. 2015; Vuong et al. 2016), as illustrated in Fig. 3a.

PTBP1 is expressed at high levels in neural stem cells and neural progenitor cells (NPC) but, upon neuronal differentiation, it becomes repressed, allowing the induction of PTBP2 expression, which in turn promotes NPC differentiation in postmitotic neurons (Li et al. 2014). In fact, it was shown that PTBP1 depletion in fibroblasts is enough to drive them towards a neuronal phenotype (Xue et al. 2013). Once the neurons become mature, the expression of PTBP2 is reduced, giving rise to an adult neuronal splicing programme (Linares et al. 2015). Together, the PTBP1 and PTBP2 interplay is thought to be responsible for 25% of neuron-specific AS events (Zaghlool et al. 2014) by coordinating splicing programmes through the use of a large set of target exons that display a range of responsiveness dependent on their levels of expression (Keppetipola et al. 2012; Linares et al. 2015). For instance, PTBP1 represses *PBX1* (pre-B-cell leukaemia homeobox 1) exon 7, which creates an embryonic stem cell form of PBX1 that does not affect neuronal genes (Linares et al. 2015). Once PTBP1 is removed, the exon is included forming a *PBX1* isoform able to promote the activation of neural genes. Another target of PTBP1 is *PTBP2* exon 10, which is skipped when PTBP1 is expressed, producing a *PTBP2* isoform that is degraded by nonsense-mediated decay (Spellman et al. 2007).

As mentioned above, the brain-specific SRRM4 also plays an important role during neurogenesis. Also known as nSR100, it targets several brain-specific exons in genes that are critical for nervous system development (Calarco et al. 2009). In fact, one of the most important roles of SRRM4 is the negative regulation of a transcriptional repressor of genes required for neurogenesis (REST). It promotes AS of REST transcripts to produce the REST4 isoform that has a reduced repressive activity, thus activating expression of REST targets in neural cells (Raj et al. 2011; Norris and Calarco 2012). Moreover, nSR100 directly outcompetes widespread neural exon repression by PTBP1 during early stages of neurogenesis (Raj et al. 2014).

Once the neuronal cell fate commitment is achieved, neurons and axons can migrate in a coordinated time and space manner to generate the laminar structure of the brain (Iijima et al. 2016). Two families of RBPs, NOVA (i.e. NOVA1 and NOVA2) and RBFOX (i.e. RBFOX1, RBFOX2, and RBFOX3), were linked to this neuronal development stage. NOVA1 is exclusively expressed in the subcortical regions and in postmitotic neurons of the central nervous system whereas NOVA2 is primarily expressed in the neocortex (Yano et al. 2010). In general, *NOVA* was shown to be important for the neuronal migration of mitotic progenitors and differentiated interneurons in the spinal cord as well as for axon outgrowth and guidance (Leggere et al. 2016). Moreover, NOVA2 was displayed as being important for cortical lamination since its absence causes the abnormal inclusion of the exon 7b and 7c in the *DAB1* transcript, a component of the Reelin pathway that controls cortical neuronal migration and lamination (Yano et al. 2010; Norris and Calarco 2012) (see Fig. 3b).

Regarding the RBFOX family, RBFOX1 is expressed in neurons, heart and muscle and its absence in mouse, especially of isoform Rbfox1-iso2, causes defects during corticogenesis due to impairments in migration, axon growth and dendrite development of excitatory neurons (Hamada et al. 2015). RBFOX2, besides being expressed in all the aforementioned tissues, is also expressed in the embryo, hematopoietic stem cells and embryonic stem cells (ESCs) and plays a more critical role in the development of the cerebellum (Gehman et al. 2012). In fact, the absence of *RBFOX2* affects the cerebellum by reducing its size and causing loss of foliation (Gehman et al. 2012). Moreover, RBFOX3 has been shown to be exclusively expressed in neurons and important for the promotion of neuronal differentiation of postmitotic neurons (Kim et al. 2013). Indeed, one of RBFOX3 targets is *Numb,* a crucial gene for the central nervous system (CNS) development since its loss of function in mice promotes deficiency in cranial neural tube closure and premature neuron production in the forebrain (Zhong et al. 2000; Kim et al. 2013). RBFOX1 is also responsible for the downregulation of RBFOX2 expression in RBFOX3-expressing cells (Dredge and Jensen 2011; Lin et al. 2016).

After the differentiation and migration processes are accomplished, neurons undergo a long period of formation and maturation of synapses. AS regulates crucial presynaptic cell-adhesion proteins for this stage named neurexins that are essential for synapse formation and transmission (Treutlein et al. 2014). This regulation is performed through the use of KHDRBS2 (KH-domain-containing, RNA-binding, signal-transduction-associated protein 2) (Iijima et al. 2014) (see Fig. 3c).

Other RBPs have also been shown to be important for synapse maturation. For instance, PTBP1 and PTBP2 are involved in synaptic maturation (Li et al. 2014) by regulating the expression of a scaffolding protein, PSD-95, that plays a key role during the synaptic maturation and plasticity of excitatory neurons (Zheng et al. 2012). NOVA seems to be equally relevant for the maturation of synapses as it regulates exons from genes that encode for neurotransmitter receptors or proteins that regulate the neurotransmitters release (Ule et al. 2005) (see Fig. 3c). ELAVL, MBNL, RBFOX1 and RBFOX3 are also reported as being important for the regulation of synaptic function (Wang et al. 2015c; Vuong et al. 2016; Lara-Pezzi et al. 2016). All this evidence supports the crucial role of AS in the different stages of the neuronal development in providing molecular tools necessary for the complex activity of the central nervous system.

Consistently with the described importance of AS in brain development and function, AS impairments are already known to be involved in several neurological diseases (Chabot and Shkreta 2016). Irimia and colleagues showed that most neuronal microexons (3–27 nucleotides) are misregulated in autism spectrum disorder and that this misregulation is linked to the downregulation of SRRM4 (Irimia et al. 2014). ELAVL2 was also shown to regulate transcripts related to autism (Berto et al. 2016). Mutations in *RBFOX1* were likewise linked to autism, as well as with mental retardation and epilepsy (Mills and Michal 2012; Lee et al. 2016). Moreover, widespread alterations in splicing patterns of ion channel genes were linked to epilepsy and Alzheimer disease (Heinzen et al. 2007). In fact, AS was also shown to be playing a role in neurodegenerative disorders. Mutations in two RNA/DNA-binding proteins, TDP-43 and FUS/TLS, were found to be related with amyotrophic lateral sclerosis and frontotemporal lobar degeneration (Polymenidou et al. 2012; Cookson 2017). However, their role in these diseases seems to be complex and is not completely clear. Alzheimer’s disease-relevant genes, such as *APP*, *TAU* or *APOE4*, are known to undergo AS (Love et al. 2015) and shifts in the ratio of different types of *SNCA* isoforms are thought to play a role in Parkinson’s disease pathogenesis (La Cognata et al. 2015). Other splicing-related, not directly causative genes implicated in Parkinson’s disease, such as SRRM2, showed likewise condition-specific alterations in splicing regulation (La Cognata et al. 2015).

The frequent association of RNA regulatory dysfunction with neurological disorders demonstrate the relevance of AS in the nervous system (Nussbacher et al. 2015). However, its function as well as the mechanisms that underlie the regulation of splicing therein are still not fully elucidated. It is, therefore, necessary to expand our knowledge on those areas to improve therapies and diagnostic methods for neurological diseases.

## Alternative splicing in gametogenesis

Spermatogenesis represents a continuous androgen-dependent developmental process defined by extensive transcriptional activity and reprogramming which is highly influenced by the interaction between germ and somatic cells. This unique regulatory mechanism guarantees faithful transition of spermatogonial stem cells throughout the meiosis process to produce haploid spermatocytes, as well as their subsequent differentiation into round spermatids and finally into functional spermatozoa.

In agreement with the notion that substantial modifications occur in the regulation of gene expression during this process, AS has been shown to be a predominant phenomenon in the testis. In fact, brain and testis are the anatomic sites where the highest levels of exon skipping events and the most specific expression of splicing-related genes take place (Yeo et al. 2004; Grosso et al. 2008; Barbosa-Morais et al. 2012). This testis-specific signature was observed in human, chimpanzee and mouse and includes the organ-specific expression of several splicing regulators such as SF3A2, SRPK1, SRPK2, as well as core snRNP components.

However, a considerable number of splicing events in human testis are not conserved in other closely related organisms and many of them account for non-functional protein products by introducing premature stop codons in transcripts’ sequences. Based on these observations, it has been proposed that part of the testis-specific splicing may represent “background” noise induced by high levels of cell proliferation, decrease of quality control or unspecific fluctuations in the expression of splicing regulators (Elliott and Grellscheid 2006). Nevertheless, several lines of evidence provide support for a relevant contribution of splicing regulation in spermatogenesis and fertility.

A classical example is the splicing-dependent reversal of the transcription factor CREM from a transcriptional repressor in premeiotic germ cells to a potent transcriptional activator in the pachytene spermatocyte stage (Foulkes et al. 1992). This functional switch regulates the expression of genes related to the differentiation of mature spermatozoa and, concordantly, infertile male patients with round spermatid maturation arrest express only the repressor version of CREM in the testis (Peri and Serio 2014). Further analyses have revealed that AS also plays critical roles during specific stages of sperm cell maturation, such as the biogenesis of the acrosome, an exocytotic vesicle present on the apical surface of the sperm head that is essential for the fusion with the oocyte plasma membrane. Acrosome formation is modulated by the two variants of proacrosin-binding protein ACRBP, the wild-type ACRBP-W and the intron 5-retaining splice variant ACRBP-V5, which are generated by AS of the *Acrbp* gene (Kanemori et al. 2013). A study in mouse epididymal sperm showed that ACRBP-V5 participates in the formation of the acrosomal granule into the centre of the acrosomal vesicle during early spermiogenesis, whereas ACRBP-W maintains proacrosin as an enzymatically inactive zymogen in the acrosome until acrosomal exocytosis in later stages (Kanemori et al. 2016). Moreover, it was recently shown that splice variants of the fibroblast growth factor receptors (FGFRs), known to regulate cell migration via PI3 K/Akt and MAPK/ERK signalling (Pintucci et al. 2002; Francavilla et al. 2013), are expressed in human testis and localise to the acrosomal region and the flagellum (Saucedo et al. 2015). Importantly, FGFRs shown activation in response to the FGF2 ligand, revealed by increased flagellar FGFR phosphorylation, which appeared associated with the activation of extracellular signal-regulated kinase ERK and Akt signalling pathways, as well as to increased sperm motility and sperm kinematics. It is therein hypothesised that FGF2, known to be present in the endometrium, the oviduct and in the oocyte vicinity (Malamitsi-Puchner et al. 2001), could bind to FGFR splice variants in the sperm acrosome to regulate fertilisation-related events.

A recent RNA-seq study evidenced a prominent reprogramming of the splicing environment during male meiosis in mice, identifying more than a hundred splicing switches, including skipping of exon 2 in the ODF2/Cenexin transcript, and mutually exclusive exons in the *Ate1* gene (Schmid et al. 2013). ODF2 has been involved in a functional switch as microtubules organiser, moving from the centriole in somatic cells to the sperm tail in post-meiotic cells, whereas *Ate1* encodes for a histone methyltransferase proposed to have important physiological roles in spermiogenic chromatin remodelling (Lambrot et al. 2012). Global changes in the levels of splicing regulators were also observed during spermatogenesis in this and other studies, including the upregulation of germ cell-specific Sam68, T-STAR, hnRNPGT, and RBMY proteins (Vernet and Artzt 1997; Venables et al. 2000, 2004; Paronetto et al. 2006) as well as alterations in the expression of non-germ cell-specific splicing factors, such as the downregulation of PTBP1, MBNL1, MBNL2, and hnRNPA1, and the upregulation of PTBP2/nPTB, BCAS2/SPF27, Tra2b, and the CUGBP ELAV-like proteins CELF1 (previously shown to be essential for normal spermatogenesis in mice) and CELF2 (Kress et al. 2007; Lambrot et al. 2012; Schmid et al. 2013; Liu et al. 2017).

The finding that members of the CELF protein group, including CELF1 and CELF2, were upregulated, while muscleblind proteins MBNL1 and MBNL2 appeared transcriptionally repressed during meiosis seems to be in agreement with the previously described antagonistic activity of CELF and muscleblind proteins (Kalsotra et al. 2008; Wang et al. 2015a; Solana et al. 2016). Moreover, the authors speculate that PTBP2 may functionally replace PTBP1 during meiosis, similar to what has been observed during neurogenesis (Boutz et al. 2007; Licatalosi et al. 2012), and suggested a suchlike replacement strategy of RBMX with RBMXL2/hnRNPGT. Consistently with these findings, an isoform-level expression profiling of genes located at the azoospermia factor (AZF) region at the Y chromosome identified 11 novel transcripts involved in human male infertility, including RBMX2, RBMXL1-1, and RBMXL1-2 (Ahmadi Rastegar et al. 2015). The same study proposed a diagnostic splicing-related signature that can be potentially used to effectively discriminate between premeiotic maturation arrest, Sertoli-cell-only syndrome, nonobstructive azoospermia, and normal testicular tissues, highlighting the importance of exploring spliced variants of candidate genes in spermatogenic failure.

RBM5 was also recently identified as a novel male germ cell splicing factor required for spermatid differentiation and male fertility (O’Bryan et al. 2013; Bao et al. 2014). A missense mutation in the second RNA recognition motif (RRM) of RBM5 appeared to induce shifts in its isoform ratios, as well as production of novelly spliced transcripts in putative RMB5 target genes, including members of the aforementioned MAPK/ERK signalling pathway (Xia and Yan Cheng 2005). Mutant mice exhibited an azoospermia phenotype (no sperm in the ejaculate) due to spermatid differentiation arrest, germ cell sloughing and apoptosis.

The growing interest in the AS regulatory mechanisms during spermatogenesis has allowed the identification of previously uncharacterised splicing-related proteins, which appeared involved in the disruption of round spermatid differentiation and male sterility. The testis-specific mammalian BET gene *Brdt* for instance, known to function as a transcriptional regulator, was associated with the modulation of gene expression as part of the splicing machinery, through the regulation of 3′-UTR processing in round spermatids in mice (Berkovits et al. 2012). Similarly, RANBP9, a member of the Ran-binding protein family (RanBP) involved in nucleocytoplasmic transport, was also found to associate in mice with numerous splicing factors including SF3B3, hnRNPM, PABPC1, and PABPC2, and has been involved in the AS of more than 2,300 mRNAs in spermatocytes and round spermatids (Bao et al. 2014).

**Fig. 4:**
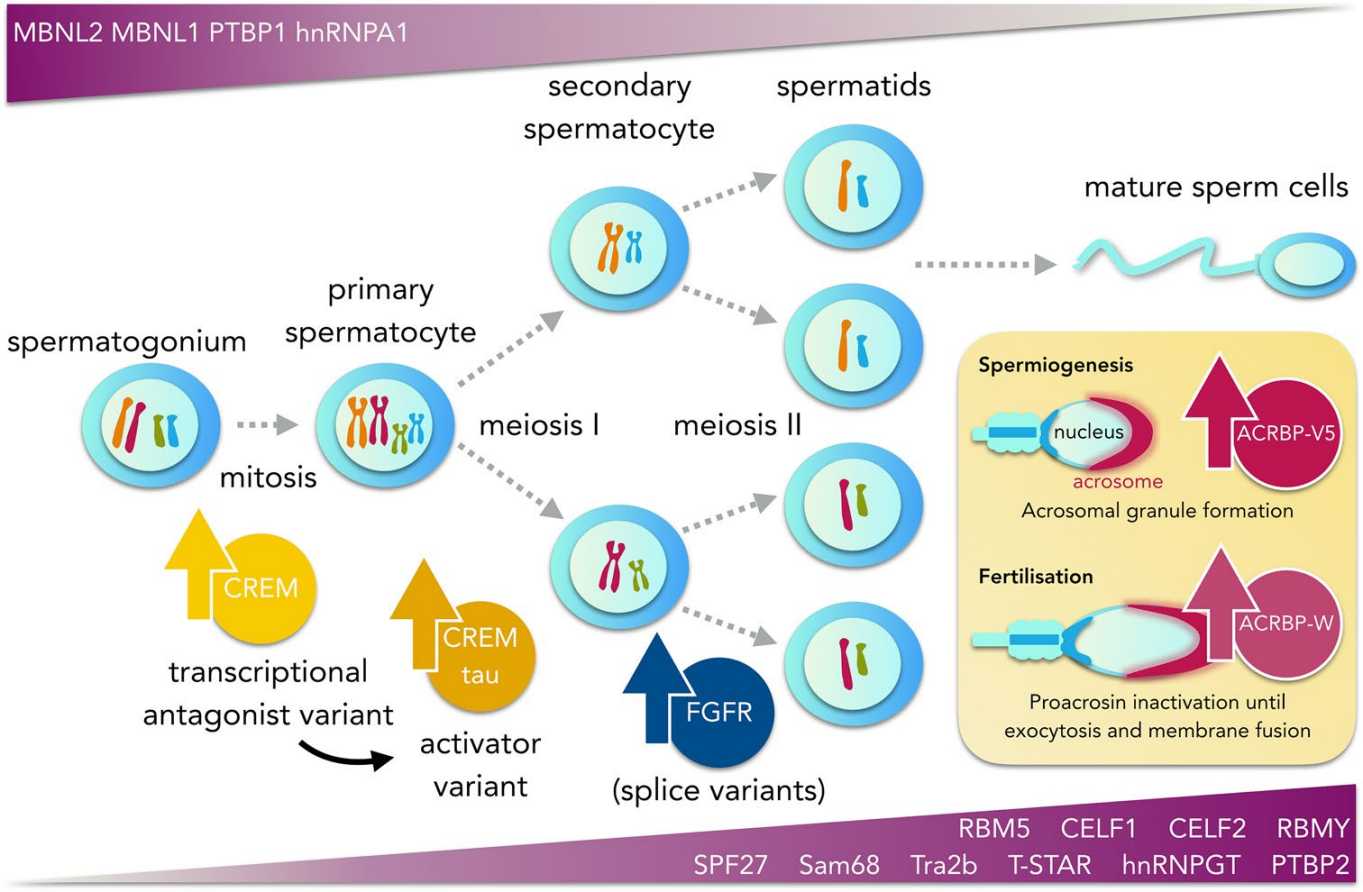
Graphical representation of spermatogenesis and its associated AS program. Temporal expression of key splicing factors and splice variants during meiotic division and spermatid maturation is represented by *violet gradients*. *Bottom gradient panel* shows the upregulation of the non-germ cell-specific splicing factors SPF27, RBM5, PTBP2, Tra2b, CELF1, and CELF2 and the germ cell-specific splicing factors (Sam68, T-STAR, hnRNPGT, and RBMY). *Top gradient panel* shows downregulation of the splicing factors PTBP1, MBNL1, MBNL2, and hnRNPA1. AS of the mRNA of the transcription factor CREM induces a functional switch from a transcriptional repressor in premeiotic cells to a transcriptional activator in the pachytene spermatocyte stage. Studies in mouse suggest that two splice variants of the proacrosin-binding protein ACRBP, ACRBP-V5, and ACRBP-W, participate in transport/packaging of proacrosin into acrosomal granules during spermiogenesis and in the promotion of acrosin release from the acrosome during acrosomal exocytosis, respectively. Similarly, splice variants of the fibroblast growth factor receptors (FGFRs) are expressed in spermatocytes and round spermatids and localise to the acrosomal region and the flagellum of mature sperm cells in humans

Interestingly, a recent study has revealed an interplay between AS regulation and higher order chromatin organisation during spermiogenesis, based on the analysis of the chromatin-binding protein MRG15 (Iwamori et al. 2016). Human MRG15 is known to recruit PTBP1 to intronic splicing silencer elements near exons through its binding to methylated H3K36 (Luco et al. 2010). In mouse spermatids, MRG15 was found also to colocalise with PTBP1 and PTBP2 at H3K36me3 sites and conditional knock-out males lacking MRG15 showed spermatogenic arrest at the round spermatid stage, concomitant with an increase in intron retention and exon skipping events, suggesting that MRG15 may be a key regulator of splicing during spermiogenesis. To note, haploid spermatids experience a profound reorganisation and compaction of their chromatin, where a histone-based nucleosomal structure is extensively substituted by a protamine-based structure, a process that requires incorporation of testis-specific histone variants, post-translational histone modifications, chromatin-remodelling complexes, and transient formation of DNA breaks. Thus, the finding that AS may be coupled to histone dynamics during round spermatid stage leads to the proposal that regulation of pre-mRNA splicing by histone modifications can be an important conceptual element to understand spermatogenesis and epigenetic disorders in male infertile patients. In Fig. 4, a graphical representation of spermatogenesis and the associated AS program described in this section is depicted.

Finally, the role of AS in human infertility may not be restricted to the spermatogenesis process. The androgen receptor (AR), for instance, is a steroid receptor transcription factor playing important roles in human reproduction. Multiple AR AS variants have been involved in androgen insensitivity syndrome and associated male infertility (Dehm and Tindall 2011; Iwamori et al. 2016), but also in polycystic ovary syndrome, one of the most common causes of female infertility (Wang et al. 2015b). The mammalian follicle-stimulating hormone receptor (FSHR) gene encodes distinct splice variants resulting from exon skipping events that correlate with low response to ovarian stimulation with exogenous follicle-stimulating hormone (FSH) (Karakaya et al. 2014). These data suggest that alterations in the AS programme regulating hormone receptor pathways may be an important pathogenic mechanism in infertility. Further studies are required not only to validate in humans the abundant evidence for AS modulation observed during mouse spermatogenesis, but also to comprehensively characterise the global splicing regulatory mechanisms governing human germ cell differentiation and reproduction.

## Alternative splicing in muscular tissues

**Fig. 5:**
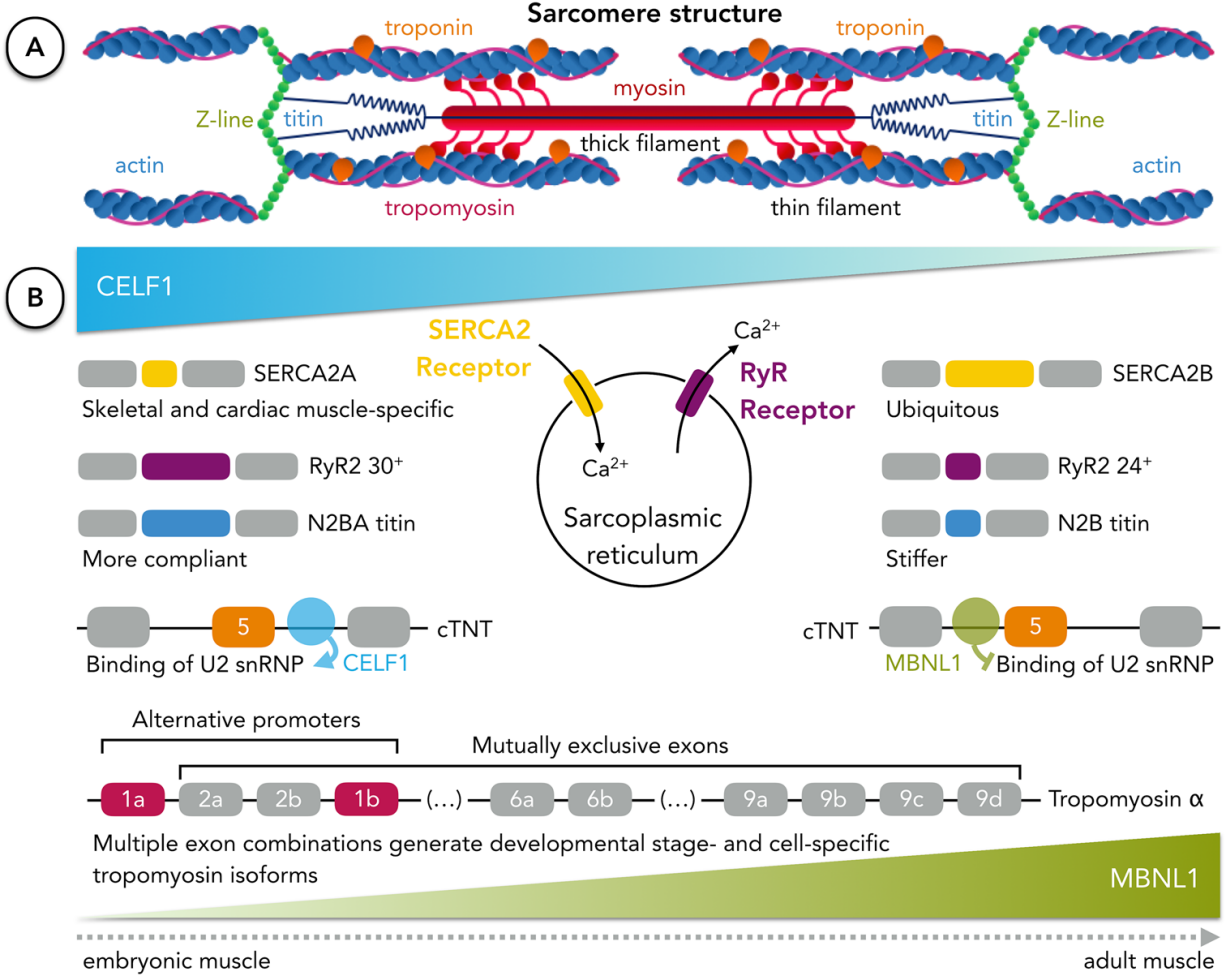
Alternative splicing of sarcomeric and membrane receptor proteins tunes muscular function. **a** Muscle contraction is achieved through the sliding between thin (rich in actin) and thick (rich in myosin) myofilaments of the sarcomere, shortening its length. Diversity of isoforms of sarcomeric proteins (such as titin, tropomyosin or troponin) required for tissue- or developmental stage-specific functions in muscular tissues arises by alternative splicing (sarcomere structure based on (Seeley et al. 2006)). **b** RNA-binding proteins MBNL1 and CELF1 are two major regulators of muscle-specific AS whose levels shift during the transition from embryonic to mature tissue. The calcium equilibrium needed for contraction of muscle cells is achieved by the coordinated activities of Ca^2+^ receptors at the membrane of the sarcoplasmic reticulum. Developmentally regulated AS of the sarcoplasmic/endoplasmic reticulum ATPase Ca^2+^ transporting (SERCA2) and ryanodine receptors (RyR) shapes calcium handling, controlling sarcomere contraction. Titin isoforms with different levels of stiffness change their relative abundance ratio in muscle cells during the transition from embryonic to adult tissue, altering myocardial compliance. The levels of the larger and more compliant titin isoform N2BA decrease with development, while the smaller and stiffer isoform N2B levels increase in mature and healthy muscle tissue. Troponin, one of the thin filament proteins, tunes the interactions between actin and myosin. MBNL1 and CELF1 regulate the inclusion of exon 5 of the cardiac troponin (*cTNT*) pre-mRNA by binding in the upstream or downstream intron, respectively. Tissue and developmental stage specificity of tropomyosin is achieved through the usage of alternative promoters and mutually exclusive exons of three of the four tropomyosin mammalian genes. In the case of the tropomyosin α gene, two alternative first exons and three sets of mutually exclusive exons contribute to the variability of tropomyosin isoforms

The functional unit of myofibrils in striated muscular tissues is the sarcomere (Fig. 5a), a complex structure formed of overlapping protein filaments, whose dynamic sliding enables the shortening of the muscle fibre, ensuring contraction (Seeley et al. 2006; Squire 2016). Several studies reported that AS may play a fundamental role on the massive transcriptomic remodelling required during the transition from embryonic to adult muscle and for the dynamic functions required for contractile proteins in sarcomeres to achieve the demands of muscular tissues, such as contraction and force generation (Kalsotra et al. 2008; Giudice et al. 2014; Wang et al. 2016).

Muscle was one of the first tissues reported to have a specific pattern of AS (Llorian and Smith 2011). MBNL and CELF protein families have been consistently described as regulating muscle-specific AS events. MBNL1 typically modulates AS in muscle by repressing or promoting the inclusion of exons when binding to their upstream introns or downstream introns, respectively (Goers et al. 2010; Barash et al. 2010; Llorian and Smith 2011). The AS pattern during muscle development is regulated, among other factors, by an antagonism between the increased levels of MBNL1 and decreased expression of CELF1 (Pistoni et al. 2010) (see Fig. 5). In fact, a study with transgenic mice replicating the embryonic expression levels of CELF1 and MBNL1 in adult heart reproduces most of the embryonic splicing profile (Kalsotra et al. 2008). Other RBPs have been reported to regulate muscle-specific AS, such as RBFOX1 and polypyrimidine tract binding proteins, and other pairs of protein families with antagonistic functions in AS regulation have been established, such as CELF and PTB (Charlet et al. 2002; Sureau et al. 2011; Llorian and Smith 2011; Lara-Pezzi et al. 2013).

Most of the reported AS-associated alterations in muscular diseases are related to the loss of adult AS programmes and mimicking of the embryonic/developing splicing profile, which is incompatible with function of developed tissues, namely, in the heart (Ho et al. 2004; Lee and Cooper 2009; Giudice et al. 2014). The postnatal development of the vertebrate heart involves extensive physiological changes to cope with the requirements of its mature function and AS has been reported to greatly contribute to the associated transcriptomic alterations (Kalsotra et al. 2008; Giudice et al. 2014). Moreover, AS is known to contribute to heart function in the regulation of important processes such as calcium handling and sarcomere contraction (Lara-Pezzi et al. 2013).

The complexity of muscular tissues arises from the diversity required for the function of sarcomeric contractile proteins. In the sarcomere, myosin thick myofilaments are able to cross-link with actin thin myofilaments upon binding of Ca^2+^ to binding sites in troponin molecules, coupled to thin filaments. The increase in Ca^2+^ levels is triggered by an action potential at the neuromuscular junction that is propagated to the whole cell through the sarcolemma into T-tubules, structures that ultimately lead to the release of Ca^2+^ by the sarcoplasmic reticulum. Accurate Ca^2+^ balance is required for concerted contraction and, therefore, of utmost importance for correct muscular function. Calcium handling is also dependent on the ion channels that enable its uptake to the sarcoplasm from the sarcoplasmic reticulum (Seeley et al. 2006). The transition from embryonic to adult cardiac muscle tissue is accompanied by isoform shifts in many of the proteins involved in the sarcomere function and the excitation–contraction coupling (Lara-Pezzi et al. 2013; Zhu et al. 2016).

Thin filament proteins, such as cardiac troponin T (cTNT), undergo AS under the regulation of MBNL1 and CELF2. In adult cardiac muscle, an increased number of MBNL1 protein molecules bind to the upstream intron of cTNT, inhibiting the binding of essential spliceosomal components, leading to the skipping of exon 5 (Warf et al. 2009). In embryonic cardiac muscle, on the contrary, inclusion of exon 5 of cTNT is enhanced by the action of CELF2 in promoting and stabilising the binding of the spliceosomal component U2 snRNP, after binding to the downstream intron (Goo and Cooper 2009). Isoform diversity of tropomyosin (another thin filament protein) is expanded by the use of alternative promoters and mutually exclusive exons from the four tropomyosin genes (Tropomyosin α, β, γ, and δ), tuning actin/myosin interaction in sarcomeres in a developmental stage- and cell-specific manner (Gunning et al. 2005; Lara-Pezzi et al. 2013) (Fig. 5b).

The balance of Ca^2+^ inside the muscle fibre is controlled by a tight orchestration of membrane receptors’ function. One of the processes involved in muscle contraction is the release of Ca^2+^ from the sarcoplasmic reticulum through the ryanodine receptors (RyR). Two developmentally regulated alternatively spliced variants of the human cardiac RyR receptor (RYR2) have been reported to affect cardiomyocyte susceptibility to undergo apoptosis by differential regulation of nuclear and cytoplasmic Ca^2+^ release (George et al. 2007). The sarcoplasmic/endoplasmic reticulum ATPase Ca^2+^ transporting, SERCA2, is responsible for pumping Ca^2+^ back into the sarcoplasmic reticulum to achieve muscle relaxation. This calcium pump has been reported to have a cardiac and slow skeletal muscle-specific isoform, SERCA2a, and the switch to the ubiquitous isoform, SERCA2b, leads to impairment of the contractile function of the heart in mice (Ver Heyen et al. 2001) (Fig. 5b).

Titin is a giant sarcomeric protein responsible for the generation of passive tension by binding to myosin and myosin-binding protein C, enabling muscle flexibility and extensibility. Titin is known to undergo AS involving its 364 exons (Gigli et al. 2016; Zhu et al. 2016), and although a great number of titin isoforms can be generated, the adult cardiac muscle expresses two classes, whose ratios define the stiffness provided to the cardiomyocyte. The N2BA titin isoform is larger and contains additional spring elements that provide lower passive tension and more compliance to the cardiomyocyte, while the N2B isoform is smaller and stiffer, comprising 60–70% of adult human cardiac titin (Gigli et al. 2016). Moreover, AS of the titin gene has been linked to the regulatory activity of RNA binding motif protein 20 (RBM20), described as a regulator of cardiac AS and whose mutations have been associated with human dilated cardiomyopathy (Guo et al. 2012; Maatz et al. 2014; Zhu et al. 2016). Alterations in titin isoform balance were found during development of rat cardiac muscle, with N2BA levels decreasing and N2B levels increasing after birth (Opitz et al. 2004; Zhu et al. 2016). Also, a study on the expression of cardiac titin in patients with dilated cardiomyopathy reported alterations at the isoform ratio level favouring the more compliant N2BA isoform, with a consequent decrease in passive myocardial stiffness (Nagueh et al. 2004; Gigli et al. 2016) (Fig. 5b).

In both ends of the sarcomere, actin filaments are attached to a filamentous, proteic disc called the Z-line (Seeley et al. 2006). The LIM domain-binding protein 3 (LDB3) plays a role in muscle function by promoting sarcomere Z-line stability during contraction and its developmentally regulated isoforms are cardiac or skeletal muscle-specific (Cheng et al. 2011; Zhu et al. 2016). Also, a recent study focusing on splicing transitions from embryonic to adult muscle involved evaluating the effect of *CELF1* re-expression in adult mouse cardiomyocytes and reported AS alteration in trafficking genes from adult to fetal patterns, resulting in multiple cardiac defects, namely, at the levels of T-tubule function, leading to impairment of the excitation–contraction coupling, calcium balance and force generation (Giudice et al. 2014, 2016).

Myotonic dystrophy (DM) encompasses a group of genetically determined multisystemic disorders that compromise skeletal muscle function and are the most common cause of muscular dystrophy (Lee and Cooper 2009; Pistoni et al. 2010). Myotonic dystrophy type I (DM1) leads to progressive weakness, cardiac conduction defects and insulin resistance, being characterised by a repeated CTG sequence in the 3′ UTR of the dystrophia myotonica protein kinase (*DMPK*) gene concomitant with massive alterations in AS patterns. The RNA resulting from the repeated CTG sequences forms a double-stranded hairpin structure in vitro that resembles the binding sites for some RBPs and sequestrate MBNL proteins to nuclear foci, accompanied by an increase in CELF1, which together contribute to the disruption of the normal adult muscle AS pattern, with embryonic-specific muscle isoforms being produced instead (Pistoni et al. 2010; Llorian and Smith 2011). Some of the alterations in the splicing programme in DM1 are related to its symptoms, such as the aberrant splicing of the skeletal muscle-specific chloride channel 1 (*CLCN1*), containing a premature STOP codon that leads to its downregulation in association to myotonia, or the increase of skipping of exon 11 of insulin receptor 1 (*IR1*), leading to insulin resistance (Savkur et al. 2001; Mankodi et al. 2002; Lee and Cooper 2009; Pistoni et al. 2010).

Moreover, an RNA-seq study on postnatal AS transitions during heart development performed in mouse cardiomyocytes and cardiac fibroblasts reported that most alterations occurred before postnatal day 28, with an enrichment of AS transitions in genes related to vesicular trafficking and membrane alterations. This is consistent with the early life acquirement of an appropriate heart function, associated with proper membrane organisation, including correct ion channel functioning and ligand uptake, contributing to correct excitation/contraction coupling. Also, a substantial fraction of the AS events related to these transitions were enriched in binding motifs for CELF1, suggesting a direct mechanism for postnatal cardiac splicing regulation. To test the hypothesis of CELF1-regulated AS having a role in the assembly of the excitation–contraction apparatus, namely, in the invagination of the T-tubules, Giudice and colleagues induced re-expression of CELF1 in adult animals which was found to trigger important alterations in cardiac function in three different tests, with the T-tubule structure mimicking the one from postnatal days 10–15 (Giudice et al. 2014).

The RNA-binding protein RBM24 has been recently identified as a regulator of a large number of muscle-specific AS events. Its inactivation in mouse led to severe malfunctions and deaths between embryonic days 12.5 and 14.5 with great loss in sarcomeres of cardiomyocytes (Yang et al. 2014). The expression of RBM24 regulated muscle-specific AS by binding to an intronic splicing enhancer in the vicinity of target muscle-specific exons, overcoming their repression by other splicing factors. RBFOX1 has been reported to co-regulate, together with MBNL1, muscle-specific AS (Klinck et al. 2014; Conboy 2016). RBFOX protein family deregulations have been associated with cardiac diseases, with decreased expression levels of RBFOX1 found in human and mouse heart failure (Gao et al. 2016). Also, Wei and colleagues recently showed, in rodents, that transverse aortic constriction, modelling compensation and posterior decompensation mechanisms involved in heart failure, leads to decreased levels of RBFOX2, with consequent splicing alterations, suggesting it may function as a pressure overload sensor (Wei et al. 2015). RBFOX1 and RBFOX2 are also involved in the regulation of AS events in Myocyte Enhancer Factor 2D (*Mef2D*) in mouse, switching the ubiquitous isoform to the one that activates the late muscle gene expression programme during myogenesis (Runfola et al. 2015).

Alternative splicing plays a major role in the specificity of striated muscle function and cardiac and skeletal muscle disorders have been associated with altered isoform ratios or global loss of the adult muscle-specific AS profiles. However, knowledge on the processes that lead the identified patterns is still poor and further research on the modes of interaction of the different AS regulators shown to have a muscle-specific activity may provide new insights into these mechanisms, with potential relevant clinical applications.

## Alternative splicing in the immune system

The immune system is composed of cells and molecules responsible for protection from infectious diseases and comprises both a rapid and general response, innate immunity, and a more specific response, adaptive immunity, which develops as a response to infection. AS has been shown to contribute for the fine-tuning of both responses. For instance, Toll-like receptor signalling pathways, involved in innate immunity, are regulated by AS and alternative polyadenylation, as reviewed in (Carpenter et al. 2014). As for adaptive immunity, AS plays a crucial role in ensuring the needed diversity and flexibility, as will be discussed in this section. The main effectors of the adaptive immune response are lymphocytes, which can be divided in two main groups: B cells and T cells. While the former are responsible for the production of antibodies, which recognise microbial antigens and both neutralise them and mark them for destruction, the latter promote the destruction of intra- and extracellular microbes and help B cells in antibody production (Abbas et al. 2014).

It is essential that lymphocytes correctly distinguish between host and pathogens, reacting only to pathogenic antigens. Tolerance to self-antigens is referred to as self-tolerance and is assured by elimination or receptor editing of self-reactive lymphocytes, mainly during maturation. Rearrangement of T-cell receptor (TCR) or immunoglobulin (*IG*) locus genes and somatic hypermutation of the immunoglobulin variable region result in a vast repertoire of receptors, which are later exposed to self-antigens to eliminate or modify self-reactive ones. Medullary thymic epithelial cells (mTECs) are responsible for self-antigen presentation to immature T cells and possess the unique ability of expressing a large fraction of all the self-antigens of the host, including tissue-specific ones (Derbinski et al. 2001). This promiscuous expression is controlled by the autoimmune regulator (AIRE) protein (Anderson et al. 2002) and has been shown to be further expanded by AS, as well as by RNA editing. mTECs were shown to have more alternatively spliced genes and express at least as many splice junctions per gene for most genes, including tissue-specific ones, as all other tissues and cell types analysed (Danan-Gotthold et al. 2016). Indeed, while the diversity generated by AS throughout host tissues poses a challenge to the immune system, it seems to be extensively used by mTECs in an attempt to comprehensively represent all the host’s self-antigens.

After maturation, AS also plays an important role in lymphocyte activation. Both B- and T-cell activations result in extensive changes to gene expression and AS. It has been suggested that transcription factors controlled by BLIMP1 repress the B-cell specific and activate the plasma cell-specific gene expression programme, promoting maturation of B cells into antibody-releasing plasma cells (Turner et al. 1994; Shaffer et al. 2002; Minnich et al. 2016). Even though AS changes in these cells are less clearly understood, a recent study, profiling the transcriptional response of B cells to activating stimuli, indicates that AS, namely, alternative exon usage, affects a wide range of genes, with an enrichment in those with signalling and receptor functions (Zhang et al. 2016). A classic example of such splicing changes can be found in the AS of the Ig heavy chain, encoded by the *IGH* locus, at the 3′ end, resulting in two distinct isoforms: a membrane-bound antigen receptor and a secreted antibody (Rogers 1980; Early et al. 1980b), the latter being more expressed upon B-cell activation (Melchers and Andersson 1973; Lamson 1984). More recently, hnRNPL-like (hnRNPLL) has been shown to directly associate with *IGH* mRNA and to be more expressed in plasma cells than in B cells, along with Elongation Factor for RNA-Polymerase II, ELL2 (Benson et al. 2012). HuR, a splicing factor encoded by *ELAVL1*, has also been shown to play a role in the splicing of several hundreds of transcripts, mainly those involved in glycolysis, the citric acid (TCA) cycle and oxidative phosphorylation, all pathways upregulated following B-cell activation (DeMicco et al. 2015; Diaz-Muñoz et al. 2015). Furthermore, Diaz-Muñoz and colleagues show that HuR is needed for B-cell proliferation and differentiation into plasma and memory B cells, as well as class-switching to produce antibodies other than IgM and IgD (Diaz-Muñoz et al. 2015).

**Fig. 6:**
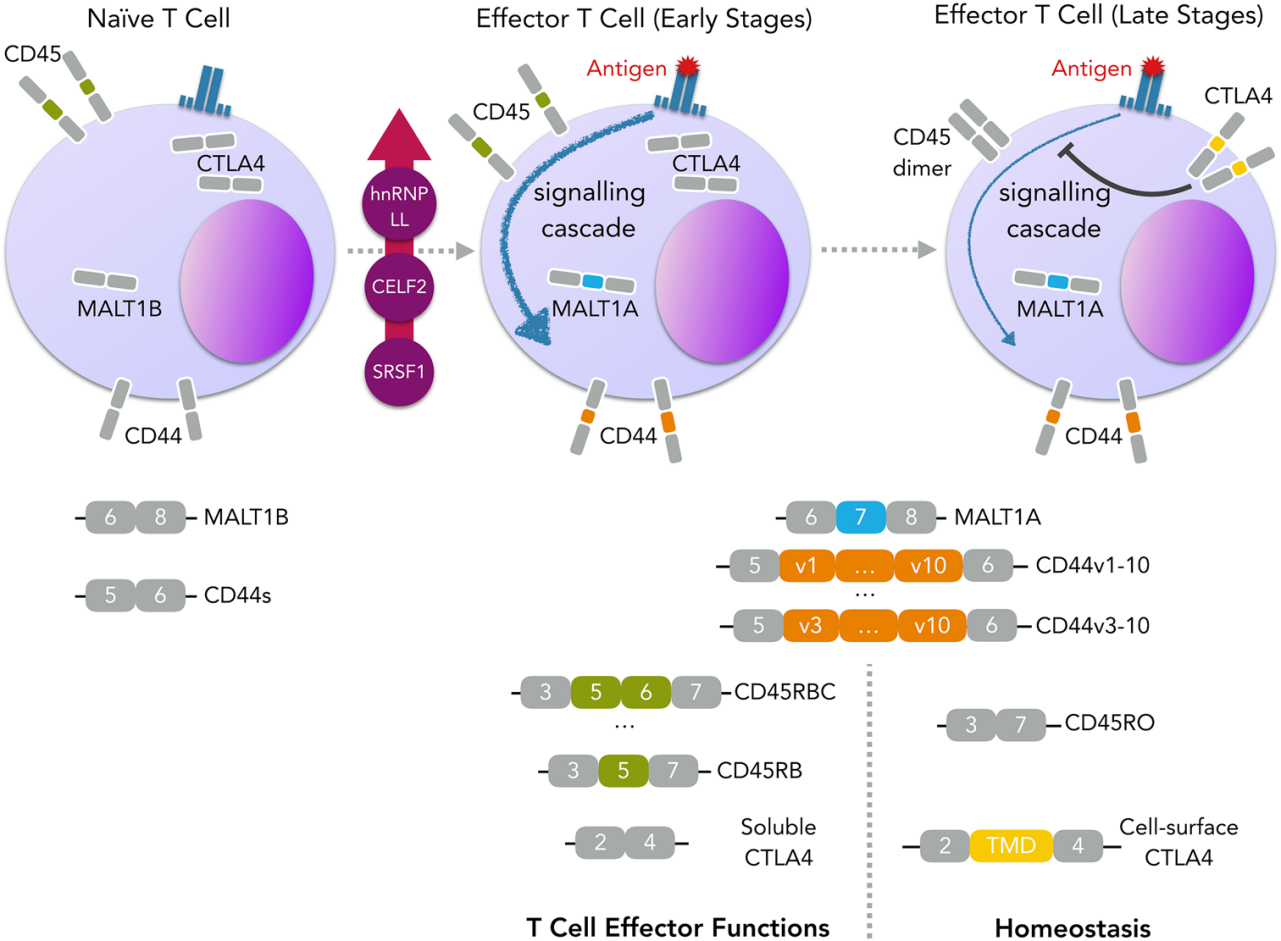
Isoform shifts following T-cell activation. T-cell activation upon antigen recognition leads to global changes in AS, from which the inclusion of MALT1 exon 7 and CD44 variable exons are highlighted. In the case of MALT1, inclusion of TRAF-binding domains contained in its exon 7 leads to a higher recruitment of TRAF6 to the CARMA1-BCL10-MALT1 (CBM) signalling complex, which facilitates IKK activation (Meininger et al. 2016). This results in an enhancement of signalling pathways downstream of TCR signalling and promotion of T-cell activation. As for the transmembrane glycoprotein CD44, ten variable exons are located in the extracellular domain of the protein, which can be excluded or included in different combinations, leading to differences in binding affinity to extracellular matrix components, namely, hyaluronic acid (Naor et al. 1997). While in resting T cells the CD44 variable exons are skipped (isoform CD44s), these are included upon activation (Arch et al. 1992). Even though the importance of this event is not yet clear, CD44 is known to be involved in T-cell homing (DeGrendele et al. 1997) and survival (Baaten et al. 2010). Later stage changes in alternative pre-mRNA splicing often impact genes involved in homeostasis and immunologic memory, from which we take CD45 and CTLA4 as examples. Skipping of alternative exons 4–6 of CD45, results in the production of an isoform more prone to dimerisation, which inhibits the role of CD45 in TCR-signalling transduction. CTLA4, on the other hand, competes with CD28 for ligand binding (van der Merwe et al. 1997), and delivers inhibitory signals that counteract the co-stimulatory signal conferred by CD28 (Krummel and Allison 1995). Upon activation, CTLA4 expression is increased and exon 3, encoding a transmembrane domain, is included (Oaks et al. 2000), drastically increasing the expression of CTLA on the cell surface and empowering the T-cell inhibitory signal. *TCR* T-cell receptor, *TMD* transmembrane domain

T cells also undergo global changes in the AS programme upon activation (Martinez et al. 2012), which start by affecting genes involved in T-cell effector functions and later genes that are relevant for homeostasis and immunologic memory (Ip et al. 2007), as shown in Fig. 6. Early AS changes affect, for instance, the *CD44* gene (Arch et al. 1992), encoding for a cell-adhesion molecule involved in T-cell homing (DeGrendele et al. 1997) or, as recently discovered, *MALT1*, responsible for channeling TCR signalling to the IKK/NF-κB signalling pathway. Inclusion of *MALT1* exon 7, containing a TRAF6-binding domain that renders the protein more active, is negatively regulated by hnRNPU and induced by TCR signalling (Meininger et al. 2016). An increase in the expression of splicing regulators upon T-cell activation has also been reported, such as hnRNP LL (Topp et al. 2008), CELF2 (Mallory et al. 2011) or SRSF1 (Moulton and Tsokos 2010), as well as phosphorylation of several splicing factors (Mayya et al. 2009). Some later stage splicing alterations act as feedback mechanisms, avoiding hyperactivity of the immune response and ensuring homeostasis, as is the case of *CTLA4* (Magistrelli et al. 1999) and *PTPRC* transcripts. *PTPRC* encodes the transmembrane tyrosine phosphatase CD45, critical for TCR signal transduction and, therefore, T-cell activation (Trowbridge and Thomas 1994). A long isoform is expressed in naïve T cells, containing either one or two of the alternative exons 4, 5, and 6, but, upon activation, *PTPRC* splicing is altered and isoforms containing none of the alternative exons are expressed (Beverley et al. 1988, 1992; Merkenschlager and Beverley 1989). These shorter isoforms are more prone to dimerisation (Xu and Weiss 2002), inhibiting CD45 phosphatase activity and resulting in lower TCR signal transduction. In naïve T cells, splicing of *PTPRC* is regulated by the hnRNPL (Rothrock et al. 2005) and SRSF1 proteins (Motta-Mena et al. 2010), while upon T-cell activation, hnRNPLL is expressed and induces repression of exons 4 (Oberdoerffer et al. 2008; Topp et al. 2008) and 6 (Preussner et al. 2012). Furthermore, PTB-associated splicing factor (PSF) is phosphorylated upon T-cell activation and represses the three variable exons (Melton et al. 2007).

In addition to the mentioned feedback mechanisms, immune activity is controlled by other mechanisms, such as apoptotic cell death, to avoid autoimmunity and assure T-cell homeostasis. The roles played by apoptosis include elimination of autoreactive T cells during maturation in the thymus and peripheral organs (central and peripheral T-cell tolerance), elimination of T cells activated for long in peripheral organs and also termination of the immune response (Abbas et al. 2014). Several genes involved in apoptosis are alternatively spliced, such as *FAS*, which encodes a death receptor. Skipping of its exon 6, containing the transmembrane domain, leads to the production of a soluble protein; inclusion of that exon results in a membrane receptor that can trigger signalling pathways leading to cell death (Hughes and Crispe 1995). This exon skipping event is regulated by TIA-1 and TIAR hnRNPs in a feed-forward mechanism (Izquierdo et al. 2005; Izquierdo and Valcárcel 2007). The importance of apoptotic regulation in the immune system is highlighted by the fact that higher levels of the soluble, anti-apoptotic *FAS* isoform are detected in patients with systemic lupus erythematosus (SLE) and mice injected with this isoform display autoimmune diseases (Cheng et al. 1994).

Deregulation of splicing events that are necessary for normal function of the immune system often leads to a wide range of diseases, from which we highlight autoimmune diseases. These usually result from defective self-tolerance or regulation, due to impaired deletion of autoreactive lymphocytes, or low numbers of cells that regulate the immune response, such as regulatory T cells (Abbas et al. 2014).

Several SNPs affecting genes with relevant roles in the immune system have been described as leading to aberrant splicing patterns. An SNP in exon 4 of the aforesaid *PTPRC* gene, encoding for CD45, leads to the inclusion of that exon by disturbing an exonic splicing silencer. While the isoform lacking variable exons is expressed in activated T cells, to regulate activity, this polymorphism resulting in increased expression of the longer isoform is one of those linked to multiple sclerosis (MS) (Lynch and Weiss 2001; Evsyukova et al. 2010). Several other genes involved in immune system function have SNPs linked to autoimmune diseases. For instance, an SNP affecting a branch point site in the *BANK1* gene, encoding for a protein involved in B-cell receptor signalling, induces skipping of the constitutive exon 2 and has been linked to SLE (Kozyrev et al. 2008). Other reports have linked skipping of exon 9 of protein-tyrosine phosphatase sigma (*PTPRS*) to ulcerative colitis (Muise et al. 2007) and reduced splicing efficiency of intron 1 of inositol 1,4,5-trisphosphate 3-kinase C (*ITPKC*) to Kawasaki disease (Onouchi et al. 2008).

Interestingly, AS has also been proposed to generate epitopes to which the organism has not been tolerized. Central tolerance only covers a set of isoforms but, in autoimmunity-prone conditions, expression of the remaining, non-tolerized isoforms increases, which may trigger an immune response (Ng et al. 2004). One example is the myelin proteolipid protein (PLP), present in either a longer isoform or a shorter one, lacking exon 3B. PLP is expressed in the thymus, but this expression is restricted to the short isoform, so the host does not acquire tolerance to exon 3B. In pathogenic conditions, damage is exerted to myelin and the longer isoform is exposed, which may trigger an autoimmune reaction that contributes to MS (Klein et al. 2000).

Alternative splicing has been shown to provide an extra layer of regulation in the immune system, from the reprogramming of B and T cells upon activation to the generation of the diversity that characterises this complex and dynamic system. Disruption of this regulatory layer by SNPs can lead to diseases, particularly to autoimmunity, which underscores the importance of unveiling new mechanisms and alterations to splicing regulation in the context of the immune system.

## Alternative splicing and transcriptomic crosstalk with human microbiota

Infectious diseases are one of the main causes of mortality worldwide and the problem increases with the development of drug-resistant pathogens. A comprehensive understanding of the underlying mechanisms of infections, particularly the molecular interactions between the host and the infectious agents, is critical to the identification of novel virulence factors and host–response pathways essential to assess and design more effective diagnostic and therapeutic strategies.

During the course of an infection, pathogens subvert cellular mechanisms of the host organisms for replication and survival, while infected host cells respond through a cascade of changes at the transcriptomic and metabolic levels. Interestingly, the recent finding of “nucleomodulins”, bacterial proteins able to act directly in the nucleus of host cells (Bierne and Cossart 2012), indicates that those pathogenic microorganisms have evolved mechanisms to actively manipulate nuclear regulatory pathways and reprogramme host gene expression to their advantage.

Moreover, emerging evidence suggests that host manipulation by pathogens may involve alterations in the AS programme of infected cells. Using an integrated approach of stable isotope labelling with aminoacids in cell culture (SILAC), 2-DE gels and matrix-assisted laser desorption/ionisation (MALDI) mass spectrometry analyses, Holland and colleagues (Holland et al. 2011) discovered significant changes in the phosphoproteome of gastric epithelial cells upon infection with the Gram-negative bacterium *Helicobacter pylori*, which causes chronic inflammation of the human gastric mucosa. Interestingly, almost one-third of the identified proteins appeared associated with the spliceosome or RNA splicing and several SR proteins exhibited alterations in phosphorylation and/or abundance. These results lead the authors to speculate that modifications in cellular splicing patterns associated with *H. pylori* infection could be the cause of changes in activity and specificity of cellular regulators such as kinases and tumour suppressors, thereby contributing to cellular dysfunction and transformation. Bacterial interaction with components of the AS machinery in host cells also occurs upon infection with Shigella, a highly adapted human pathogen that causes bacillary dysentery. Shigella’s invasion within epithelial cells involves the delivery of a subset of effectors directly into the cytoplasm of host cells using a complex bacterial structure called type III secretion. One of these protein effectors is IpaH9.8, shown to translocate into and accumulate within the nucleus, where it disrupts splicing activity upon binding to the U2AF35 splicing factor and reduces the expression of chemokines and cytokines involved in neutrophil recruitment and proinflammatory responses (Okuda et al. 2005). Based on these observations, it was proposed that the role of IpaH9.8 in bacterial infection is to modulate the acute innate immune response through the regulation of RNA synthesis, thus promoting efficient colonisation within the host cells. Orthologs of IpaH9.8 have also appeared to translocate into the nucleus of host cells, such as SspH1 of *Salmonella enterica* (the major cause of salmonellosis) and YopM of *Yersinia pestis* (responsible for plague) (Haraga and Miller 2003; Benabdillah et al. 2004), although their association to the host’s transcriptional regulation and AS programmes is still to be elucidated.

Viruses are other examples of how pathogens can take advantage of the splicing machinery in the infected cells for their own benefit. It has been shown that adenovirus, HIV and herpesvirus depend on host splicing modulators for viral RNA processing (Muhlemann et al. 2000; Fukuhara et al. 2006; Nojima et al. 2009). Moreover, viral proteins can modulate splicing of cellular pre-mRNAs that in turn regulate virus propagation. Herpes simplex virus type 2 (HSV-2), for instance, was found to modify the expression of promyelocytic leukaemia (PML) isoforms in host cells through the activity of ICP27 (Nojima et al. 2009), a viral protein known to interact and colocalise with cellular splicing regulators such as SR proteins, snRNPs and other spliceosome components (Sandri-Goldin and Hibbard 1996; Bryant et al. 2001; Sciabica et al. 2003). Furthermore, ICP27 has been involved in the splicing regulation of viral genes critical for pathogenesis through modulation of intron retention events (Sedlackova and Rice 2008). Further analyses revealed that, in fact, ICP27 also induces intron retention within the PML transcript upon binding to the 3′ splice site of intron 7a, resulting in an isoform switching from PML-II to PML-V, which in turn affected HSV-2 replication. As PML has been proposed to contribute to intrinsic antiviral defence but also to promote efficient viral propagation (Chee et al. 2003; Ching et al. 2005), PML isoform switch was proposed to potentially contribute to the mechanisms controlling these antagonistic functions of PML in the host response to viral infection. A recent RNA-seq analysis of the HSV-1 infected host transcriptome of human primary fibroblast BJ cells revealed profound changes in both gene expression and AS in host cells (Hu et al. 2016). Several splicing factors, such as PABPC1, YBX1, XAB2, and ZFP36, were shown to be upregulated and more than a thousand AS events appeared dysregulated, including events that contribute to the activation of the cellular stress response. However, it is still unclear how exactly HSV-1 infection led to changes in cellular AS processes. To note, about 22% of the alternatively spliced events identified in this study correspond to intron retention events. It is possible that at least part of these alterations can be due to the activity of ICP27, as HSV-1 ICP27 has also been shown to promote intron retention in infected cells (Sedlackova and Rice 2008). Importantly, the transcriptomic analysis of HSV-1-infected cells revealed perturbations not only at the level of AS, but also in alternative polyadenylation and general isoform composition, suggesting that the viral modulation of host RNA processing is more extensive and may be, as in the case of transcriptional regulation, a critical component of the complex pathogen-host molecular interactions.

Several lines of evidence suggest that tumourigenesis caused by tumour viruses is also mediated by cellular and viral AS programmes. Some known tumour-promoting protein isoforms are produced through AS of viral oncogene transcripts (Zheng 2010; Young et al. 2011; Ajiro and Zheng 2015) and numerous cellular mechanisms have been shown to modulate this oncogenic viral splicing (Wang and Manley 1995; Rosenberger et al. 2010; McFarlane et al. 2015; Graham and Faizo 2016). More recently, RNA-seq combined with de novo transcriptome assembly in cultured cells infected with the oncogenic human papillomavirus HPV16 revealed the upregulation, in infected cells, of the splicing factor CELF3 as well as several differentially expressed novel human transcripts which appeared associated with well-known cellular pathways altered in cancer such as the MAPK and the VEGF signalling (Xu et al. 2016). Based on this evidence, it has been proposed that viral AS represents a promising therapeutic target in the treatment of viral-induced tumours, such as in the context of other non-tumourigenic viral infections where therapeutic strategies targeting the splicing machinery have already been tested (Hernandez-Lopez and Graham 2012).

**Fig. 7:**
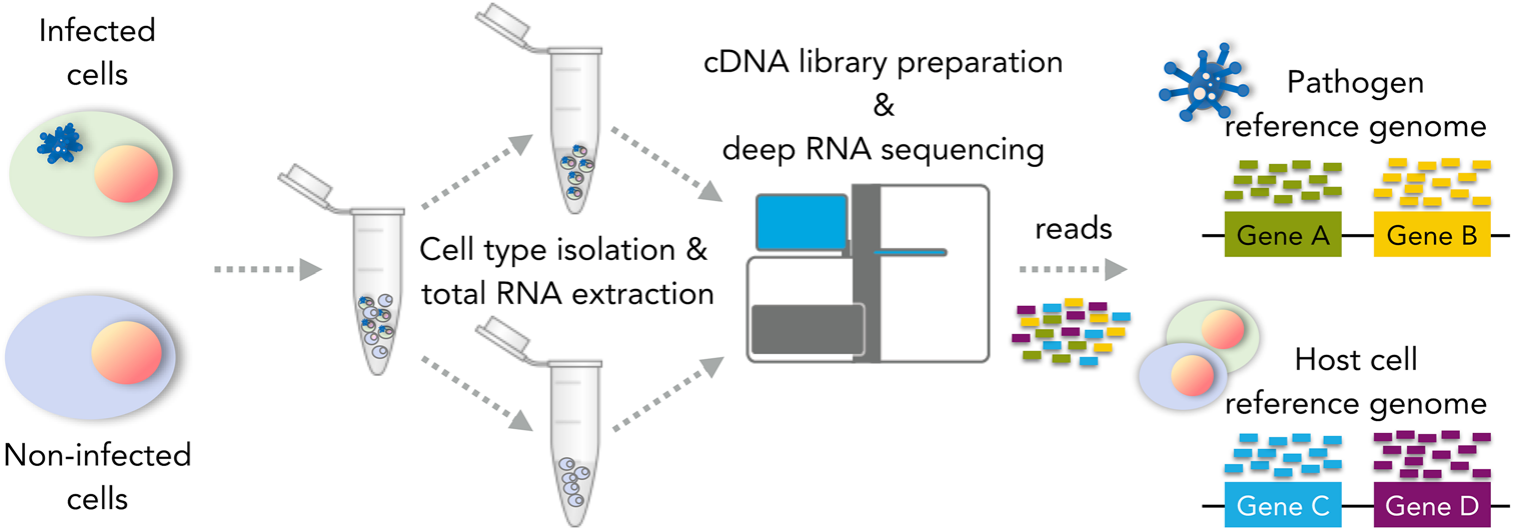
Dual RNA-seq workflow. Due to the relative difference in total RNA abundance between host cells and pathogen in most infection models, deep sequencing is required to obtain a more precise profiling of transcriptomic changes associated with the infection process. This strategy allows the transcriptomic analysis of both host and pathogen at different time points during infection, with the discrimination between the two taking place only at the bioinformatics stage

Traditionally, the analysis of gene expression patterns in host cells and pathogens has been carried out using microarrays or reverse transcription PCR of a single species at a time. However, the advent of systems biology methods that integrate multi-omic data is transforming our understanding of infectious diseases by considering the host–pathogen molecular interface as a unified module. Among these novel approaches, the development of the dual RNA sequencing (dual RNA-seq) technique has allowed the simultaneous analysis of transcriptomes of host cells and intracellular pathogens (Hernandez-Lopez and Graham 2012; Westermann et al. 2012; Rosani et al. 2015; Aprianto et al. 2016) (see Fig. 7), revealing cascades of changes in gene expression but also unpredicted functions of pathogen genes in regulating other RNA-synthesis processes such as the expression of long noncoding RNAs (Westermann et al. 2016). We anticipate that dual RNA-seq analysis will be particularly important not only in the characterisation of novel regulatory mechanisms of AS and other post-transcriptional modifications during the course of infection, but also in the assessment of the contribution of human microbiota to normal physiology and disease predisposition.

## Alternative splicing in cancer

There is a growing recognition of the key role played by aberrant splicing in tumourigenesis, cancer progression and resistance to therapy. Accumulating evidence shows that dysregulated splicing is frequently associated with the inactivation of tumour suppressors and the activation of oncogenes. A recent systematic analysis of SNVs across six cancer types profiled in The Cancer Genome Atlas (TCGA) Project revealed that SNVs causing intron retention were enriched in tumour suppressors and that the vast majority of these events generated premature termination codons leading to nonsense-mediated decay, thus suggesting that intron retention is a common mechanism of tumour-suppressor inactivation (Jung et al. 2015). This notion was further validated by a TCGA transcriptomic data analysis that revealed increased levels of intron retention relative to normal tissue controls across most cancer types, suggesting that an abundance of intron-containing mRNAs in tumour cells may increase the transcriptional diversity of many cancers (Dvinge and Bradley 2015).

Aberrant splicing also participates in the activation of oncogenes by producing splice variants with novel proliferative or survival abilities. Recent genomic characterisation of different types of cancer revealed “spliceosomal mutations” that affect splice site choice as well as exon recognition motifs, which induce isoform switching or even entirely new splice variants specific to tumour cells (Harbour et al. 2013; Martin et al. 2013; Alsafadi et al. 2016). In addition, alterations in splicing regulatory mechanisms can result in the imbalanced expression of splice variants of the same gene playing antagonistic functional roles and thus the associated protein interaction network can also be affected (Boise et al. 1993; Cheng et al. 1994). Several examples of this splicing-driven functional inversion in cancer occur in genes regulating pro- and anti-apoptotic signalling, such as the case of caspase-9 expressing a short splice variant (caspase-9S) that inhibits full-length caspase-9-dependent apoptotic signalling by interfering with its binding to Apaf-1 and the formation of the so-called “apoptosome” complex (Seol and Billiar 1999), the long isoforms of BCL-X (BCL-XL) and APAF1 (APAF1L) known to inhibit programmed cell death, whereas their short isoforms (BCL-XS and APAF1S) promote it (Boise et al. 1993; Walke and Morgan 2000), the proapoptotic long isoform of caspase-2 (Casp-2L) that antagonizes the anti-apoptotic short isoform (Casp-2S) (Droin et al. 2001), and the complex pattern of pro- and anti-apoptotic isoforms of the tumour-suppressor *TP73* gene generated through the use of alternative promoters and AS (Stiewe and Pützer 2002).

**Fig. 8:**
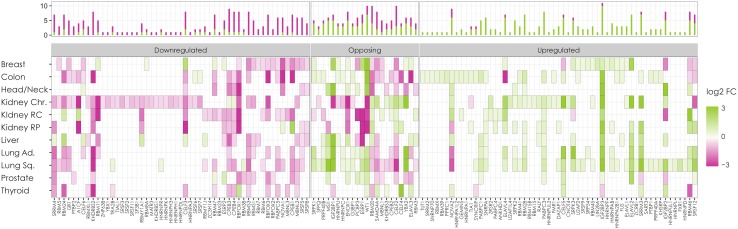
Alterations in the expression of splicing factors in cancer. A pan-cancer analysis using TCGA data revealed 132 splicing factors differentially expressed between tumour and normal samples (*x*-axis). Patterns of upregulation and downregulation across different tumor types (*y*-axis) are shown in *green* and *violet gradients,* respectively. The *color intensity* indicates the log2-fold change (log2 FC). Splicing factors are clustered into three groups according to the incidence of each expression pattern in the analysed tumours: frequently downregulated (*left*), frequently upregulated (*right*), and tendency to show an opposite pattern between the three kidney and the rest of tumor types (Opposing). The bar plot in the top indicates the frequency of tumor types with up-(*green*) or down-(*violet*) regulation for each factor. Kidney Chr, kidney chromophobe; Kidney RC, kidney renal clear cell carcinoma; Kidney RP, kidney renal papillary cell carcinoma; Lung Ad, lung adenocarcinoma; Lung Sq, lung squamous cell carcinoma. Image adapted with permission, from Sebestyén et al. (2016)

Moreover, alterations in the gene expression of multiple splicing factors and their regulators represent another level of dysregulation in the AS programme in cancer. Interestingly, the same splicing factor can appear upregulated in some cancers and downregulated in others (see Fig. 8), indicating that the regulatory pattern of its expression is likely to be distinct among tumour types. Examples of well-characterised families of splicing factors differentially expressed across several types of cancer are SR proteins, including SRSF1, SRSF3 and SRSF6 (Karni et al. 2007; Jia 2010; Anczuków et al. 2012; Jensen et al. 2014), and hnRNPs, such as hnRNPA1, hnRNPA2 and PTBP1 (David et al. 2010; Clower et al. 2010). Several important roles in cancer development have been recently found to be played by other splicing factors, such as the function of FOX2, RBM4, and CELF2 as tumour suppressors (Ramalingam et al. 2012; Wang et al. 2014; Yong et al. 2016), the oncogenic effect of CELF1 (House et al. 2015), or the regulation of SRSF3 splicing patterns by PTBP1 and PTBP2 (Guo et al. 2015). The function of these and other cancer-associated splicing factors is reviewed in detail in (Fu and Ares 2014) and (Dvinge et al. 2016).

Ultimately, altered expression of splicing factors and aberrant splicing programmes in general contribute to critical aspects of the so-called hallmarks of cancer, which represent biological capabilities acquired during tumourigenesis and cancer progression. They include sustained cell proliferation, the evasion from growth suppressors and apoptosis, the deregulation of cellular metabolism, the avoidance of immune destruction, and the activation of angiogenesis, invasiveness, and metastasis (Hanahan and Weinberg 2011). In fact, aberrant splicing itself has been proposed as a novel hallmark of cancer (Ladomery 2013).

SRSF1 splicing factor and its associated kinase SRPK1, for instance, have been implicated in the regulation of splicing events with prominent roles in several oncogenic signatures, including the retention of intron 4 in the cyclin D1b splice variant that promotes cellular transformation (Olshavsky et al. 2010), splicing events conferring escape from apoptosis such as the inclusion of *BIM* exon 3 or exon 4 in Caspase 9 (Shultz et al. 2011; Leu et al. 2012), isoform switches in the vascular endothelial growth factor (VEGF) with pro and antiangiogenic functions (Nowak et al. 2010), and inclusion of the exon 3b in the GTPase Rac1 and skipping of exon 11 in the tyrosine kinase receptor RON, both events known to induce cell dissociation, mobility and invasion in several cancer types (Ghigna et al. 2005; Gonçalves et al. 2014).

Apart from these and other well-characterised alterations in AS patterns affecting key components of cancer hallmarks, such as PT53, hTERT, EGFR, CD44, KLF6, FAM3B, MENA, NUMB, or BRAF, which have been extensively reviewed before (David and Manley 2010; Bonomi et al. 2013; Oltean and Bates 2014; Sveen et al. 2016), novel insights into tumour-associated dysregulation of splicing and its biological consequences have been recently described. One case is the functional interplay between splicing mechanisms and transcription modulation by transcription factors activity, which is now emerging as an important regulatory axis in cancer cell biology. A new general splicing-based regulation of tumour growth has been proposed based on the observation that an isoform switch of the transcription factor TEAD4 modulates the expression of components of the Hippo-YAP pathway, known as a core regulator of cell cycle, proliferation and apoptosis (Qi et al. 2016). The MYC transcription factor, overexpressed in most human cancers and associated with highly proliferative tumours and poor prognosis, was found to regulate the maintenance of a fully functional splicing machinery by controlling the transcription of snRNP components. Through this mechanism, MYC ensures proper RNA processing and consequent expression of full-length proteins that sustain cancer cell survival and proliferation such as ATR, EP400 and DVL1 during lymphomagenesis (Koh et al. 2015). To note, SRSF1 was also found to regulate MYC by promoting the inclusion of exon 12a in the tumour-suppressor BIN1, known to bind MYC and reduce its oncogenic activity (Sakamuro et al. 1996).

The link between transcriptional regulation and the splicing programme in cancer also involves modifications at the level of high-order chromatin structure. Altered function of chromatin modifiers has been implicated in the disruption of proper RNA processing and splicing, such as the case of mutations in the histone methyltransferase SETD2 in kidney tumours (Simon et al. 2014; Grosso et al. 2015). Similarly, the binding of alternatively spliced short isoforms of splicing factor SON near transcription start sites was found to inhibit mixed lineage leukaemia (MLL) complex-mediated methylation of histone H3K4, a common landmark found at promoter regions of activated genes. Importantly, those short SON isoforms appeared markedly upregulated in acute myeloid leukaemia (Kim et al. 2016) and their overexpression enhances the growing capability of hematopoietic progenitors in vitro. Based on these results, it was proposed that an increase in alternatively spliced short isoforms of SON induces aberrant transcriptional initiation in leukaemia.

Moreover, a revealing contribution of AS to hypoxia-dependent increase of genetic instability in cancer has been recently characterised (Memon et al. 2016). Conditions of low oxygen occurring within most solid tumours are associated with poor patient outcome and resistance to therapy. In the study performed by Memon et al., hypoxic colorectal cancer cells exhibited systematic alterations in AS that contributed to the control of protein levels by increasing intron retention, which in turn favoured the expression of noncoding isoforms and a rapid decline in protein synthesis. Importantly, the increase in intron retention levels was observed in genes involved in specific pathways, including those associated with DNA damage response. These results reveal that changes in isoform usage under tumour hypoxia are the consequence of a coordinated reprogramming of AS.

The recent advent of genome-wide analyses of AS events in cancer has been revealing cases of global alterations involving hundreds of differentially spliced transcripts, as well as mutations and/or alterations in the expression of components of the core splicing machinery and regulatory splicing factors. In this regard, the molecular and clinical information contained in the TCGA repository has been a valuable tool to identify differences in splicing patterns between cancer and normal samples and between different tumour molecular subtypes (Brooks et al. 2014; Anczuków et al. 2015; Ryan et al. 2016; Dominguez et al. 2016; Sebestyén et al. 2016; Shen et al. 2016). TCGA data were also used in systematic pan-cancer analyses, revealing a pool of splicing events commonly altered across different cancer types and splicing factors whose expression strongly associates to cancer-specific splicing signatures, such as that of RBFOX2, QKI, PTBP1, MBNL1/2 and CELF2 (Sebestyén et al. 2015, 2016; Tsai et al. 2015; Danan-Gotthold et al. 2015).

Given the large amount of information that TCGA and other sample collections provide, involving several types of omics data and associated clinical features, the challenge now is integrating this knowledge to identify cancer-specific core regulatory mechanisms upstream of altered splicing networks and to characterise the functional significance and cause-effect relationship between cancer-specific changes in AS and oncogenesis.

## Splicing therapy

The previous sections of this review describe how misregulation of AS, namely, through perturbation of *trans*-acting factors that can trigger widespread splicing defects and/or disruptions in *cis* that can alter splice sites and other splicing sequence regulatory elements, can have an impact in human health (Garcia-Blanco et al. 2004). Moreover, according to the latest report of the Human Gene Mutation Database, around 10% of human inherited diseases are due to single base-pair substitutions mutations located in splice sites (Stenson et al. 2009). However, this estimation does not take into account mutations in other splicing *cis*-regulatory elements nor in the actual promoter or coding sequences of *trans*-acting factors, suggesting an even more prevalent role of splicing in human genetic diseases (Ward and Cooper 2009). Based on the above evidence, several therapeutic approaches based on modulation of splicing for different human diseases are being explored nowadays.

In fact, the great structural diversity of RNA and its lack of repair mechanism enhance the impact of therapeutics targeting it (Hermann and Westhof 1998). Thus, by leveraging the dynamic nature of RNA turnover, it is possible to time limit and modify the therapy according to individual responses (Douglas and Wood 2011).

**Fig. 9:**
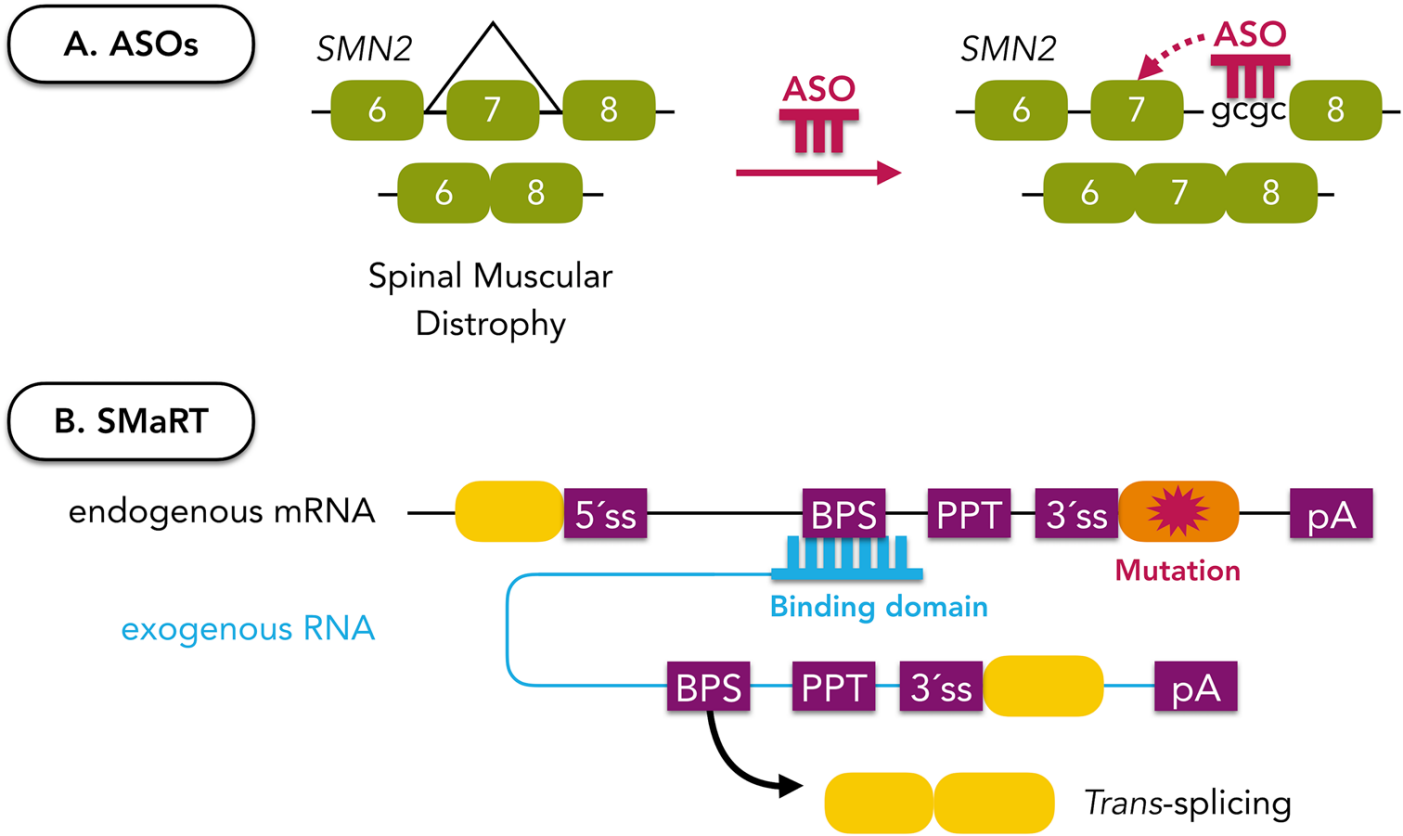
Splicing therapy methods. **a** Antisense oligonucleotides (ASOs) are being used for spinal muscular atrophy to correct the aberrant splicing of exon 7 of SMN2. The ASO binds to the unique GC-rich sequence located within the downstream intron to promote the exon 7 inclusion. **b** Spliceosome-mediated RNA *trans*-splicing (SMaRT) method relies on the correction of alterations at the post-transcriptional level by modifying the mRNA sequence. An exogenous RNA is introduced in targeted cells to induce a splicing event in *trans* with the target endogenous sequence, generating a chimeric RNA with exons from the exogenous and the endogenous RNA free of mutations. 5′ss, 5′ splice site; 3′ ss, 3′ splice site; BPS, branching point site; PPT, polypyrimidine tract; pA, polyadenylation signal

One of the approaches vastly adopted to target splicing is the use of antisense oligonucleotides (ASOs). ASOs are synthetic molecules composed of nucleotides or their analogues that bind to a nucleic acid molecule with a complementary sequence (Bauman et al. 2009). They can be used to target a splice site by blocking it and thereby alter its recognition by the spliceosome, redirecting splicing to an adjacent site (Havens and Hastings 2016). ASOs can also be used to prevent the binding of *trans*-acting regulatory splicing factors by targeting their binding sites (Havens et al. 2013; McClorey and Wood 2015). Diseases for which this therapy is being developed include spinal muscular atrophy (SMA), Duchenne Muscular Dystrophy (DMD) and amyotrophic lateral sclerosis (ALS). Most of SMA cases are linked to downregulation of the *SMN1* gene and the aberrant splicing of exon 7 of the *SMN2* gene, a nearly identical copy of *SMN1*. ASOs are being used to correct the aberrant splicing of exon 7 of *SMN2* and promote its inclusion by binding to the unique GC-rich sequence located within the downstream intron, as illustrated in Fig. 9a (Singh et al. 2009; Osman et al. 2016). DMD cases are associated with mutations in the *DMD* gene that frequently cause a deletion-induced frameshift in exon 51 by creating a premature termination codon that will produce a truncated and usually non-functional dystrophin protein (Havens and Hastings 2016). This commonly DMD-associated deletion can be restored using ASOs to induce skipping of exon 51 (Scotti and Swanson 2016). As for ALS, ASOs were designed to lower the mRNA levels of *SOD1*, whose mutation is responsible for 13% of familial ALS cases, by intrathecal administration and went through a phase I clinical trial (Miller et al. 2013). ASO therapies for SMA and DMD are also on clinical trials (Fletcher et al. 2017; Aartsma-Rus and Krieg 2016).

Another method being developed for these disorders as well as others, such as cystic fibrosis and Huntington’s disease, is SMaRT (Spliceosome-mediated RNA *trans*-splicing). It also relies on the correction of alterations at the post-transcriptional level by modifying the mRNA sequence through the introduction of an exogenous RNA in targeted cells to induce a splice event in *trans* between the exogenous RNA and the target endogenous pre-mRNA (Berger et al. 2016) (see Fig. 9b). This process generates a chimeric RNA with exons from the exogenous and the endogenous RNA free of mutations.

However, ASOs and SMaRT techniques present some drawbacks. For instance, ASOs are not efficaciously delivered because they are subjected to nuclease susceptibility in circulation, leading to a short half-life (McClorey and Wood 2015). Moreover, negatively charged ones have a limited passive diffusion through the cell membranes (McClorey and Wood 2015). Nevertheless, research in the field keeps trying to find new methods to enhance ASOs’ delivery, especially in organs with difficult access such as the brain, where crossing the blood–brain barrier is a challenge. One method being explored to circumvent these problems is the use of cell-penetrating peptides (CPPs), small peptides able to carry peptides, proteins, nucleic acids and nanoparticles across the cellular membrane (Zahid and Robbins 2015). This technique made it indeed possible to enhance the correction of *ATM* aberrant splicing causing ataxia-telangiectasia, a recessive neurogenetic disorder, by being able to deliver the ASO to the brain and cerebellum (Du et al. 2011). Delivery limitations have also promoted the discovery of small molecule modulators of splicing (Salton and Misteli 2016), identified as a good spliceosome-targeting tool and that can be synthetic or derived from natural products as fungi, medicinal plants and bacteria (Martínez-Montiel et al. 2016). For instance, the spliceosome SF3b subunit has been shown to be targeted by three bacterial natural products, pladienolide, herboxidiene and the FR901464 molecule, as well as by meaymicin, a synthetic analogue of FR901464 (Albert et al. 2009; Webb et al. 2013). They are all cytotoxic agents and are mostly recognised as antitumour agents (Salton and Misteli 2016; Kumar et al. 2016). In fact, E7107, a small molecule that also targets the spliceosome, has gone to a phase I clinical trial for treating solid tumours (Eskens et al. 2013). Moreover, new small molecules can be potentially found by high-throughput screening approaches. Mandrasin, for instance, was found by screening a highly curated library of about 72,000 drug-like small molecules using a high-throughput in vitro splicing assay, being shown to have the ability of inhibiting splicing in cultured human cell lines (Pawellek et al. 2014). Most small molecule modulators of splicing affect important components of the spliceosome such as SF3b but others, known to inhibit protein acetylation and deacetylation, can affect RNA processing via stalling of spliceosome assembly (Kuhn et al. 2009; Bates et al. 2017). For instance, borrelidin, an antifungal compound, was found to have an antiangiogenic activity in tumour and binds to the splicing protein FBP21, which is one of the structural proteins of the spliceosome (Woolard et al. 2011). However, their lack of specificity and consequent potential to alter splicing of multiple unspecific genes limits their employment in therapeutic strategies (Havens et al. 2013).

Here, we have described the most prominent techniques being developed to manipulate splicing with therapeutic purpose, aware of the need for improvements at the levels of the delivery system of ASOs and the specificity of small molecules. Moreover, AS is being progressively recognised as a promising therapeutic target, highlighting the need for a more profound understanding of the splicing-related mechanisms involved in disease conditions. In this respect, the *PP1γ2*, *Nek2A*-*T*, and *NIPP1*-*T* genes are currently being investigated as alternatively spliced targets for signal transduction therapeutics in male infertility (Fardilha et al. 2004). Furthermore, in cancer, targeting components of spliceosome has also been suggested as a potential therapy. For instance, genetic or pharmacological inhibition of the spliceosome in vivo was shown to associate with an impair in survival, tumourigenicity, and metastatic proclivity of MYC-dependent cancers (Hsu et al. 2015).

The increasing knowledge about mechanisms of splicing regulation will also provide new conceptual tools to improve the already known techniques or even to create novel treatment strategies for modulating splicing in disease contexts, unveiling splicing therapy as a tool for personalized medicine.

## Concluding remarks

As once stated in Christopher Nolan’s film *The Prestige* (Nolan and Nolan 2006), “Every great magic trick consists of three parts or acts. The first part is called *The Pledge*. The magician shows you something ordinary (…).” In a cellular context, the spliceosome performs canonical intron excision. “The second act is called *The Turn*. The magician takes the ordinary something and makes it do something extraordinary.” In this case, the spliceosome is able to carry out splicing in alternative ways. “Now you’re looking for the secret… (…) That’s why every magic trick has a third act, the hardest part, the part we call *The Prestige*.” Indeed, AS has an outstanding ability to allow the expression of highly specialised condition- and tissue-specific isoforms that contribute to different essential functions in the complex human physiology.

In this review, we describe how this is performed not only by the spliceosome but also with the assistance of other splicing regulators. However, a more comprehensive understanding of how these molecules and networks interact to regulate global and tissue-specific splicing programmes is still required. It is, therefore, imperative to unveil these regulatory mechanisms to be at the forefront of molecular characterisation of tissue function and disease.
